# Inhibitory neurons exhibit high controlling ability in the cortical microconnectome

**DOI:** 10.1371/journal.pcbi.1008846

**Published:** 2021-04-08

**Authors:** Motoki Kajiwara, Ritsuki Nomura, Felix Goetze, Masanori Kawabata, Yoshikazu Isomura, Tatsuya Akutsu, Masanori Shimono

**Affiliations:** 1 Graduate Schools of Medicine, Kyoto University, Kyoto, Japan; 2 Department of Physics and Center for Complex Systems, National Central University, Taiwan, Republic of China; 3 Molecular Science and Technology Program, Taiwan International Graduate Program, National Central University and Academia Sinica, Taipei, Taiwan; 4 Department of Physiology and Cell Biology, Tokyo Medical and Dental University, Tokyo, Japan; 5 Bioinformatics Center, Institute for Chemical Research, Kyoto University, Kyoto, Japan; 6 Hakubi center, Kyoto University, Kyoto, Japan; Ghent University, BELGIUM

## Abstract

The brain is a network system in which excitatory and inhibitory neurons keep activity balanced in the highly non-random connectivity pattern of the microconnectome. It is well known that the relative percentage of inhibitory neurons is much smaller than excitatory neurons in the cortex. So, in general, how inhibitory neurons can keep the balance with the surrounding excitatory neurons is an important question. There is much accumulated knowledge about this fundamental question. This study quantitatively evaluated the relatively higher functional contribution of inhibitory neurons in terms of not only properties of individual neurons, such as firing rate, but also in terms of topological mechanisms and controlling ability on other excitatory neurons. We combined simultaneous electrical recording (~2.5 hours) of ~1000 neurons in vitro, and quantitative evaluation of neuronal interactions including excitatory-inhibitory categorization. This study accurately defined recording brain anatomical targets, such as brain regions and cortical layers, by inter-referring MRI and immunostaining recordings. The interaction networks enabled us to quantify topological influence of individual neurons, in terms of controlling ability to other neurons. Especially, the result indicated that highly influential inhibitory neurons show higher controlling ability of other neurons than excitatory neurons, and are relatively often distributed in deeper layers of the cortex. Furthermore, the neurons having high controlling ability are more effectively limited in number than central nodes of k-cores, and these neurons also participate in more clustered motifs. In summary, this study suggested that the high controlling ability of inhibitory neurons is a key mechanism to keep balance with a large number of other excitatory neurons beyond simple higher firing rate. Application of the selection method of limited important neurons would be also applicable for the ability to effectively and selectively stimulate E/I imbalanced disease states.

## Author summary

How small numbers of inhibitory neurons functionally keep balance with large numbers of excitatory neurons in the brain by controlling each other is a fundamental question. Especially, this study quantitatively evaluated a topological mechanism of interaction networks in terms of controlling abilities of individual cortical neurons to other neurons. Combination of simultaneous electrical recording of ~1000 neurons and a quantitative evaluation method of neuronal interactions including excitatory-inhibitory categories, enabled us to evaluate the influence of individual neurons not only about firing rate but also about their relative positions in the networks and controllable ability of other neurons. Especially, the result showed that inhibitory neurons have more controlling ability than excitatory neurons, and such neurons were more often observed in deep layers. Because the limited number of neurons in terms controlling ability were much smaller than neurons based on centrality measure and, of course, more directly selected neurons based on their ability to control other neurons, the selection method of important neurons will help not only to produce realistic computational models but also will help to stimulate brain to effectively treat imbalanced disease states.

## Introduction

The brain is a highly non-random network system. Inside of the system, electrical information flows through the networks in a highly balanced manner under mutual interactions between the excitatory and inhibitory neuron pools. Therefore, the extraction of the rules that exist behind the non-random complex networks of these two types of neurons is a crucial scientific topic.

The history of E/I balance is long as it relates to disease. From over two decades ago, E/I (excitatory and inhibitory) balance has been regarded as the key factor for understanding and establishing treatment not only for epilepsy [1], but also for various mental diseases such as schizophrenia [2] and autism [3, 4]. Computational studies in terms of E/I balance have also been accumulated from over the course of two decades [5]. Recently, beyond the phenomenological observations, causal influences from E/I imbalance at the medial prefrontal cortex to behavioral deficits [6] were also experimentally verified. Although we know that suppression of inhibitory neurons causes less selectivity for external stimuli [7], it is also true that E/I balance is an over-simplified representation when considering the inherently wide diversity and non-uniformity of huge numbers of neurons [4, 8].

Network theory has been widely applied to brain data, especially at a macroscopic scale, to quantify such non-random architectures, [9–11] and also has been even more widely applied beyond neuroscience [12]. When we take a closer view of the microcircuits of neurons, several previous studies demonstrated complex intertwined architectures and have also extracted comprehensive designs and their rules [13–16].

Historically, random networks were defined as networks having Erdős–Rényi graph model, in which all members have a similar opportunity to connect with other members [17]. The non-randomness beyond this fundamental work has been studied from many perspectives, even if we limit studies of neuronal microcircuits. For example, previous studies of functional connectivity patterns demonstrated the small-worldness, namely the co-existence of short path-lengths and high clustering properties in the cortex [18, 19], scale-free like topology and existence of hub neurons in the hippocampus [20, 21], log-normal distribution [22], and also structure-function relationships among convergent connections [23] and specific motifs in cerebellar network [24]. In summary, these past studies of local neuronal circuits revealed various specific non-randomness of neuronal networks and that a relatively small proportion of neurons can influence global network dynamics. Our knowledge about the difference of contributions between E/I neurons in terms of relative positions in a large population of neurons, especially relative to controlling ability of other neurons, is yet fairly limited even though the importance has been discussed in other ares of research in the field of biology [25–27], neuronal networks of *C*.*elegans* [28] and Macroconnectome [29].

This research evaluated how E/I neurons distinctly located within the effective networks of neurons by evaluation using several graph theory analyses. This analytic scheme allows us to quantitatively evaluate different roles of individual neurons in terms of the relative positions and interactions with the many other neurons. The effective network was reconstructed from neuronal spikes recorded by a cutting-edge Multi-Electrode Array System. Transfer Entropy(TE) was selected to qualify the effective networks, quantifying the direction of information being transferred, among neurons. The connectivities defined from neuronal activity can be categorized into functional connectivity and effective connectivity. Functional connectivity evaluates statistical dependence of dynamic couplings using correlation, covariance, spectral coherence, or phase lockings between two time series. Although these can change even on the short time scales evaluation does not explicitly quantify causal influences. In general, effective connectivity was developed to explicitly deal with the causal influences between or among more than two different time series [30–32]. TE has several preferred abilities for estimation ability [33], and for theoretical analyses [34–36]. Therefore, TE has been widely adopted in various neuroscientific studies. Although there are many strengths of TE, it cannot distinguish between excitatory and inhibitory connections. The current study applies a new analysis scheme solving this issue [37] to our experimental recordings of neuronal spike data. We explain in detail the new analysis scheme in the subsection “effective connectivity estimation” within the “material and method” section.

More importantly, the estimation of connectivity will not be the final destination of neuroscience. The main purpose of this study was set to quantitatively evaluate how differences of relative positions within the networks of excitatory and inhibitory neurons play different roles in terms of controlling ability of other neurons. Evaluations of connectivity patterns provide more information than simple differences of their spatial adjacency, such as participating layers. When comparing excitatory and inhibitory neurons in the cortex, it is well known that the number of excitatory neurons is much larger than inhibitory neurons. In such an environment, how can inhibitory neurons keep the balance with excitatory neurons? It is often discussed that inhibitory neurons have a higher firing rate than excitatory neurons to keep the E/I balance, however, it is not yet well-known how the relative position of inhibitory neurons helps to keep the E/I balance.

This study will answer this question by following three steps: First, we evaluate the basic statistical properties of neuronal ensembles. The fundamental property of neuronal activity is firing rate, and the fundamental property of network architecture by most is considered to be the weight distribution and the degree distribution. Our past studies demonstrated that neuronal firing rates and connectivity weights follow a log-normal distribution, and that the degree distribution shows a long-tailed form [16, 38]. We specifically test if these properties sustain or not when we observe excitatory and inhibitory neurons separately. Then, we also defined the recording cortical regions much more accurately than before by referencing these with MRIs [39].

Second, we quantified the differences of relative positions of excitatory and inhibitory neurons (E/I neurons) within the comprehensive network architecture of the microconnectome. Especially because we were interested in the relative difference of “influence” of E/I neurons to other neurons, and we evaluated how central individual neurons are based on k-core Centrality (KC).

Third, we evaluated the relative influence of excitatory or inhibitory neurons in terms of their ability to drive or control other nodes in the network system based on the Feedback Vertex Set (FVS) method. Note that FVS is a well-known concept in graph theory [40, 41], and its relationship with attractors as well as controllability to attractors were was hown in [42] and in [25], respectively. Furthermore, it was demonstrated that genes and proteins corresponding to FVS nodes tend to play important biological roles [25–27].

From these steps, we could reveal that inhibitory neurons are not only more active than excitatory neurons but also that they also have higher controlling ability of the entire network dynamics in local cortical neuronal circuits.

## Materials and methods

### Animals & ethics statement

**Fig 1:**
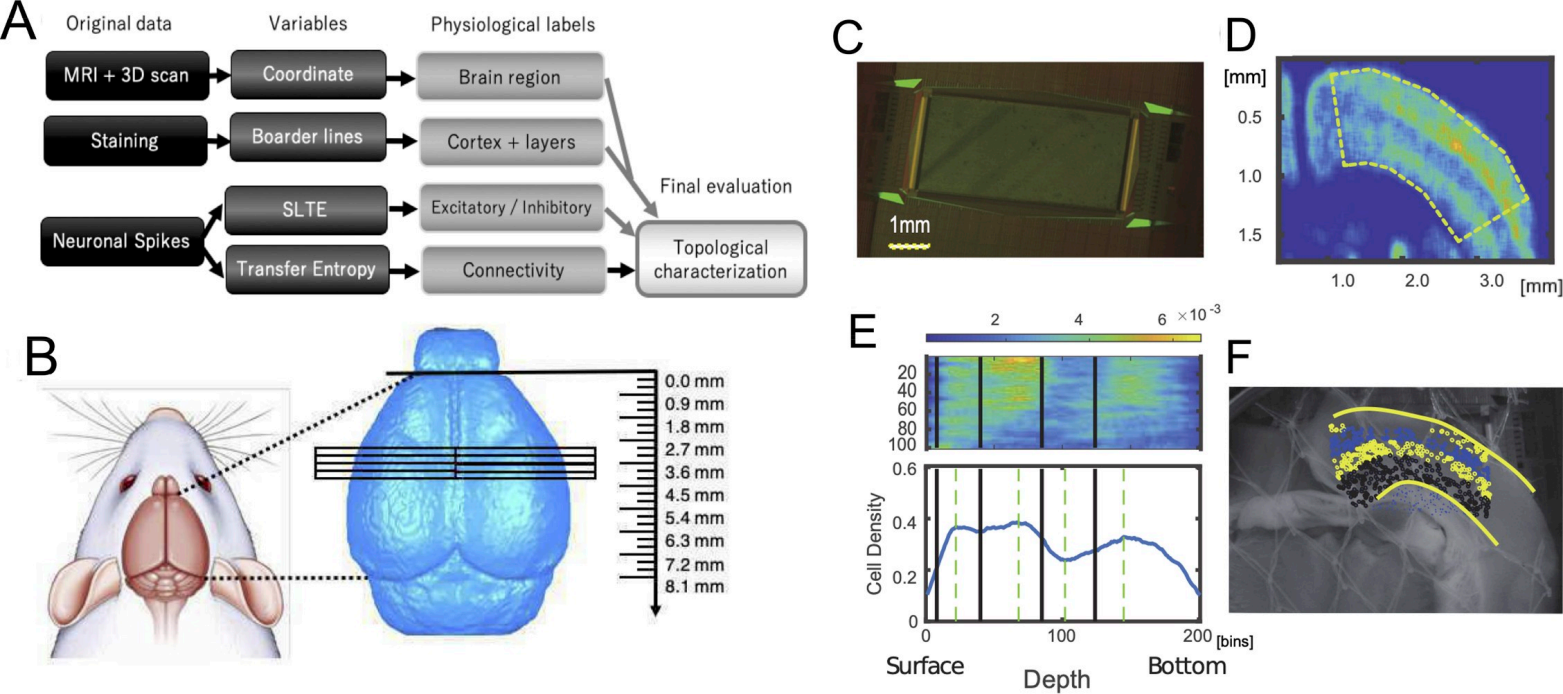
**Data process pipeline, brain regions, slices, staining and layer detections: A** Data process pipeline of the entire data process flow by four sets of data; MRI, 3D scan, Staining and Neuronal spikes. First, MRIs and 3D scans were prepared and analysed to obtain coordinates of brain slices. Second, physiological information, such as boundaries of the cortical areas within the slices, the cortical layers were defined by staining data, and excitatory and/or inhibitory cells. Third, the connectivity architectures were provided by indices of Transfer Entropy (TE). Finally, topological characteristics were calculated from the connectivity architectures while comparing them with other physiological labels and analyzed. **B** Brain regions’ boundaries are estimated through anatomical coordinates used to prepare slices from left or right cortical hemispheres. The three dimensional view of recorded brain regions is shown in S1 Fig. **C** Multi-electrode array photograph used in this study. **D** Smoothed NeuN staining image shows Non-uniformity of density of neurons. The curved regions show as dotted lines in the panel. **E** Density expressed at the panel **D** was re-shaped into a straight box as shown in the top panel at **E**, and the sum along the y-axis orthogonal to the cortical surface was expressed at the lower panel of **E**. Four layer boundaries were then determined and shown as thick lines in the lower panels being mapped onto the non-stained photographic image. **F** All neurons identified from the spike sorting process were categorized into individual layers.

We used female C57BL/6J mice (n = 7, aged 3–5 weeks; Fig 1B). All animal procedures were conducted in accordance with the guidelines of animal experiments of Kyoto University, and have been approved by the KU Animal Committee (Application ID: 20026). The mice were anesthetized with 2% isoflurane in air flowing through the box at a flow rate of 1.4 L/min using an anesthesia system, which is composed of an isoflurane vaporizer, air flow meter, and air compressor. Then, mice were located in an acrylic anesthesia box attached on a heater mater adjusted to 37°C. After checking the anesthesia is sufficient, we placed the mouse in an MRI-compatible cradle in the prone position.

### All data process pipeline

In this study, we will process three data sets (Fig 1A). The first data are magnetic resonance imaging (MRI) of the whole brain and 3D scans of the brain surface. From those data, we obtained the exact coordinates from which the slices were prepared. The second type of data was the staining data. From the staining data, information on the boundaries of cortical regions and intracortical laminar structures were obtained within the brain regions where electrical activity was measured. The final data was spike data of neural activity. From the data, we computed Transfer Entropy and the sorted local transfer entropy (SLTE) indices to obtain information on the structure of connections between neurons and the excitatory-inhibitory cell label, respectively. In particular, we analyzed the topological positioning of individual neurons given a physiological label by the data of their connection structures. In the following chapters, each of these processes will be described. All neuronal spike data is shared at a public web service ([43];https://data.mendeley.com/datasets/p8czktrz7k/1), and programming codes are shared at https://github.com/Motoki878. The code and data to reproduce the results are also available from a repository named Network Quantifications in the same github page. They are also summarized at http://shimono-u.net/codes/.

### MRI acquisition

MR experiments were conducted on a 7 T, 210 mm horizontal bore, preclinical scanner (BioSpec 70/20 USR, Bruker BioSpin MRI GmbH, Ettlingen, Germany), equipped with a gradient system of 440 mT/m at 100 μs ramp time. A quadrature volume resonator (inner diameter 35 mm, T9988; Bruker BioSpin) was used for RF excitation and signal reception. MRI data was acquired with ParaVision 5.1 software (Bruker BioSpin). Three dimensional T_2_ weighted (T2W) images of the mouse whole brain were acquired with relaxation enhancement (RARE) sequence (Fig 1B). The acquisition parameters were as follows (based on the protocol TurboRARE-3D; Bruker BioSpin): repetition time (TR), 2000 ms; echo time (TE), 9 ms; effective TE, 45 ms; RARE factor, 16; acquisition matrix size, 196 × 144 × 144; field of view (FOV), 19.6×14.4×14.4 mm^3^; acquisition bandwidth, 75 kHz; axial orientation (coronal orientation in scanner setting): fat suppression with 2.6 ms-gaussian-shaped π/2 pulse with 1051 Hz bandwidth followed by spoiler gradient; 2 dummy scans; number of averages, 3; acquisition time, 2 h 42 m. 2.59-ms excitation and 1.94-ms refocusing pulses were used. The shape of their pulses was sinc-3 shape multiplied by a gauss function with a 25% truncation level (sinc3) and their bandwidth was 2400 Hz.

### Preparation of chemical solutions

Preceding the neuronal activity recordings, we prepared two solutions, an ice-cold cutting solution (CS) and an artificial cerebrospinal fluid (ACSF). The necessary ice-cold CS was 500 mL for each experiment, and it was a mixture of the following chemical materials; 2.5 mM KCl, 1.25 mM NaH2PO4, 7 mM MgCl2, 15 mM glucose, 25 mM NaHCO3, 0.5 mM CaCl2, 11.6 mM sodium ascorbate, 3.1 mM sodium pyruvate, and 100 mM choline choline chloride, bubbled with 95% O2 and 5% CO_2_. The pH was adjusted to 7.3–7.4. We also prepared 1 L ACSF, and the content is 127 mM NaCl, 2.5 mM KCl, 1.25 mM NaH_2_PO_4_, 1 mM MgCl_2_, 15 mM glucose, 25 mM NaHCO_3_, and 2 mM CaCl_2_, bubbled with 95% and 5% CO_2_. ACSF was also warmed (~34°C) and adjusted the pH to 7.3–7.4.

### Slice preparation and electrophysiological recording

To start neuronal spike recording using an MEA system, we extracted the brain after sufficiently anesthetizing with 1%-1.5% isoflurane, and then transferring it to a Petri dish (100 mm x 20 mm) filled with the ice-cold cutting solution in which air containing 95% O_2_ and 5% CO_2_ was flowing. We made 2–5 slices (300 μm thick) on a diagonal angle to the cortical surface from the extracted brain using a vibratome (Neo Linear Slicer NLS-MT; DOSAKA EM CO., LTD). The angle was carefully selected and checked by embedding the slices onto the MRI space [39]. To optimize the cutting speed, frequency, and vibration amplitude of the system we set them as 12.7 mm/min for the speed, 87–88 Hz for the frequency, and 0.8–1.0 min for the swing width, respectively. When we temporarily needed to cut a brain carefully, we additionally slowed down the cutting speed. While cutting the brain slices, we recorded the sliced coordinates in the format, including the anterior-posterior coordinates, hemisphere, and other conditions. All slices, analyzed in this study, were taken from the dorsal cortex including the primary somato-motor area, including the barrel sensory area, diagonally to the cortical surface (Fig 1B). Then, we gently transferred the slice to a beaker filled with prewarmed (~34°C) ACSF using a thick plastic pipette, and incubated them in the beaker at ~34°C for 1 h.

After 1 h had passed, we selected one slice per animal and moved it on the chip using a thick plastic pipette, and set the position using a soft brush to properly record from specific brain regions including the cortex. The outline of the array was rectangular (2.0mm×4.0mm) and 26000 electrodes were uniformly arranged (Fig 1C), and the inter-electrode distance was 15 μm (Maxwell Biosystem, MaxOne) [44]. Preceding the main recording, we performed so-called “pre-scan” as a combination of 30[sec] recordings from densely distributed 1020 channels included in 25 groups, and selected sensors showing stronger responses than 0.1 Hz and 0.05 mV for the main recording. Then, we started the main recording of spontaneous neuronal activities from selected up to 1020 sensors for ~2.5 hours. During recording, the slices were perfused at 1mL/min with ACSF that was saturated with 95% O_2_/5% CO_2_ while controlling the temperature of the perfusate around 34°C. Notice that 2.5 hours are significantly longer than our past studies [16]. Evaluations in the computational model showed that the extension significantly improved in the following estimation performances, and the firing rate did not decay in 2.5 hours for our experimental setting.

### Brain surface scan

We recorded a 3D scan surface three different times using a 3D structured light technology scanning system (SCAN in a BOX; Open Technologies). We performed 3D scan recording at three times; The three recordings are whole brain, brain blocks after cutting into two blocks and before making slices, and brain blocks that remained after making multiple brain slices. The whole process was performed based on the *3D novel embedding overlapping (3D-NEO) protocol* [39]; The first 3D scan recording was done just after extracting a whole brain from a mouse head. Here, we removed the brain and gently dropped it into a Petri dish with ice-cold cutting solution (CS) after decapitating from a mice fully anesthetized with 1.0−1.5% isoflurane. The second recording was performed from two brain blocks cut in the middle of the coronal plane. The cut brain blocks were attached on the surface of the brain-block-base, which consisted of a 6.6 cm^2^ square and 0.5 cm^2^ thick magnet ring with sticky tape, using instant glue. We prepared the brain-block-base to transfer the brain blocks smoothly from 3D scanner to brain slicer [39]. Third 3D scan recording was performed from the remaining brain block after all slicing processes. Before scanning, we gently wiped fluid from the brain surface using the microfiber cloth, which is important to effectively absorb water in order to prevent diffuse reflections. In all scans, the rotating angle was 22.5 degrees, and we performed an automatic coregistration among images taken from 16 different angles. After scanning, we checked that all scanned surfaces are nicely overlapped with each other.

We processed the scanned images using the 3D scan and processing software IDEA (including SCAN in a BOX). First, we integrated small mismatches among 16 images using the automatic alignment option by IDEA. Then, we merged the objects of integrated images recorded from dorsal and ventral or frontal and occipital, using the manual alignment option. The optimization algorithm is the iterative closest point (ICP) algorithm without nonlinear deformation as frequently applied to hard 3D objects [45]. Finally, we made a meshed object with high resolution from the merged object by the mesh generating option, and saved it as stl-binary format to compare with MRIs of the individual brains.

### Spike sorting

We performed offline spike sorting using the SpyKING CIRCUS software. The main strength of the algorithm utilized in the software is highly accurate even though the calculation cost is low by categorizing spikes to neurons based on a template-matching method, in which templates were produced as representative waveforms averaged within a part of the time series [46]. The low computational cost was beneficial for our large number of electrodes (>1000).

First, spikes were detected as threshold crossings of a 6 standard deviation line from the high-pass filtered time series with a Butterworth filter and also whitening to delete pseudo correlation originated from external noise using 20 second data where spikes did not appear. The order of the filter was three and the cutoff frequency was 500Hz. Then, we isolated waveforms as 5 time sections around the randomly chosen peak points. Second, we performed clustering for individual groups of waveforms which were once divided into several groups located within a 150 [μm] radius This step was implemented in Spyking CIRCUS to reduce necessary computational memory. The waveform data, maximally 10000 samples, were projected into five dimensional principal component space. Third, we performed a density-based clustering [46], which gathers other samples surrounding the peaks after estimating local density peaks locating at centers of individual clusters [47]. We also iteratively merged different clusters, in which “normalized distance” is smaller than 3. Fourth, we estimated templates as two dimensional descriptions of individual clusters. The two dimensions consisted of averaged extracellular waveform and the variance. The second component was orthogonal to the first component in the sense of the, so called, Schmidt decomposition. Based on similarity with the first component, so in the waveform, of the template and the raw data, we sought individual spikes originating from the same putative neurons one by one. Fifth, we merged two neurons if the different templates were too similar (correlation > 0.975). Finally, we eliminated neurons whose firing rate was lower than 0.2Hz because there could be noisy samples.

### Immunohistochemistry staining

Immunohistochemistry staining was performed on the same day where the MEA recording is finished, Then, we fixed the slices with the 4% paraformaldehyde (PFA) in phosphate-buffered saline (PBS) overnight at 4°C. On the second day, we incubated the slice with antigen retrieval solution (10 mM sodium citrate, pH 6.0) for 20 min at 95~98°C after washing the slices 3 times with PBS for 5 min in total, and cooled the solution to the home temperature. Then, we prepared the blocking solution by adding 4% normal goat serum (NGS) and 0.5% octylphenol ethoxylate (Triton X-100) to the 1% Na bisulfite-Tris solution (pH 7.5). After washing the slices with 1% Na bisulfite in 50 mM Tris-buffered saline, we incubated the slices with the blocking solution for 2 hr at room temperature (20~25°C). We also prepared the primary antibody solution by adding 1% NGS and 0.5% Triton X-100 to the 1% Na bisulfite-Tris solution and diluting primary antibodies. Then, we incubated the slices with the primary antibody solution, consisting of 1:800 of mouse anti-GAD67 and 1:500 of rabbit anti-NeuN, overnight at 4°C. Please notice that this study used information only of NeuN staining. On the third day, we prepared the Tris-NaCl solution (8.5 g/L NaCl in 50 mM Tris-buffered saline, pH 7.5) and the secondary antibody solution by adding 3% NGS and 0.3% Triton X-100 to Tris-NaCl and diluted secondary antibodies, consisting of 1:500 of goat anti-mouse and 1:500 of goat anti-rabbit. Then, we washed the slices 3 times with the Tris-NaCl solution for 5 min in total and incubated it overnight at 4°C. On the fourth day, we washed the slices 4 times with the Tris-NaCl solution for 5 min in total. Then, we mounted the slices on slide, embedded with antifade reagent (SlowFade Gold; invitrogen), and enclosed slices with a coverslip, and recorded the distribution of cell bodies at a magnification of 10x from the slides using a fluorescence microscope (All-in-One Fluorescence Microscope BZ-X710; Keyence).

### Extracting layer categories

We applied image processing consisting of four steps to the given fluorescent images: *First*, we extracted positions of cell bodies from original NeuN staining images. *Second*, we defined distribution densities of excitatory or inhibitory cell bodies. *Third*, we defined layers based on the distributions densities. Now, we explain the two steps in detail:

*First*, we detected cell positions after performing preprocesses of staining images to reduce noise. The noise reduction step eliminates small masses of bright pixels after filling in the gaps or holes generated by random noise. The following cell detection process identified cell bodies’ positions using the Watershed algorithm. Here, we first drew contour lines that gradually rose from the surfaces of cells toward the center of cells. Using the “height” given by the contour lines, we next detected boundaries between cells overlapping each other. So, we could define centers of cells from the detected cells’ external boundaries or regions.

*Second*, we aimed to *draw boundaries of layers* based on cells’ density distributions from NeuN images. So, we first defined the density distribution by smoothing the distribution of cells as number of cells existing in 50×50 pixel regions (Fig 1D). To define boundaries of layers, we extended the curves corresponding with surface and bottom of the stained image to straight lines once. Then, we summed the number of cells in the direction perpendicular to the cortical layer to obtain one curving line of cell density histogram.

The cell density histogram typically shows three convex points and two concave points between the convex points commonly for all cortical samples (Fig 1E, bottom panel). The first convex point counted from the cortical surface indicates the center of layer 3. The second convex point locates at the middle of layer 4. The third convex point far from the cortical surface involved in the layer 6. Therefore, two concave points individually correspond to the boundary between layer 2/3 and layer 4, and the middle point of the layer 5.

*Third*, from these considerations, we defined all boundaries by the following steps; (1) between the cortical surface and the first convex point, we regarded the 1/3 from the cortical surface as the boundary between layer 1 and layer 2/3. (2) as mentioned, we defined the boundary between layer 2/3 and layer 4 at the concave point between the first and second convex point. (3) we defined the boundary between layer 4 and layer 5 at the middle between the second convex point and the second concave point, and also defined the boundary between layer 5 and layer 6 at the middle between the second concave point and the third convex point (Fig 1E, bottom panel). Owing to this systematic procedure, we could prepare boundary lines on the original staining images.

### Overlaying among images

Now, we hoped to map a set of boundaries among layers on the Multi-electrode array through two steps: *First*, we overlapped the layer divisions from staining data onto photographic images taken just after MEA recordings (Fig 1E). *Second*, we overlapped the MEA coordinates onto the photo image. These processes finally gave us layer categories for all electrically recorded neurons. We now present a more detailed explanation of the individual steps mentioned (Fig 1C). Now, we add more information about the two steps:

We simply call the photograph taken just after the electrical recording as a non-stained image. In the *first* step, to overlap the stained image and the non-stained image, we selected common typical points for them (10 points on the cortical surface and 7 points on the bottom of cortex, and other maximally other typical points). By optimally mapping each other based on the iterative closest point algorithm [48], we got a transformation matrix from the stained image to the non-stained image.

The *second step* is to overlap the non-stained image and MEA coordinates., We selected four corners in the non-stained image, and got the transformation matrix, which optimally overlap the four points between the MEA coordinate and the non-stained image. Finally, we were able to map all neurons estimated from the spike sorting process onto the non-staining image, and to give layer labels for individual neurons (Fig 1E).

### Connectivity estimation

Connectivity estimation was achieved by establishing methodology to evaluate the information transfer among different variables beyond spurious correlations or confounding factors is a crucial issue [49–52]. We selected transfer entropy (TE) to quantify the information transfer because several previous studies have demonstrated its advantages both in terms of the theoretical formulation and regarding the requirements imposed by the physiological data [35, 36, 53]. Past studies using Transfer Entropy have successfully demonstrated the structure-function relationship [54–58], consistent topology with patch-clamp experiments and existence of hubs and modules [16], and also a log-normal distribution of connectivity weights [38].

**Fig 2:**
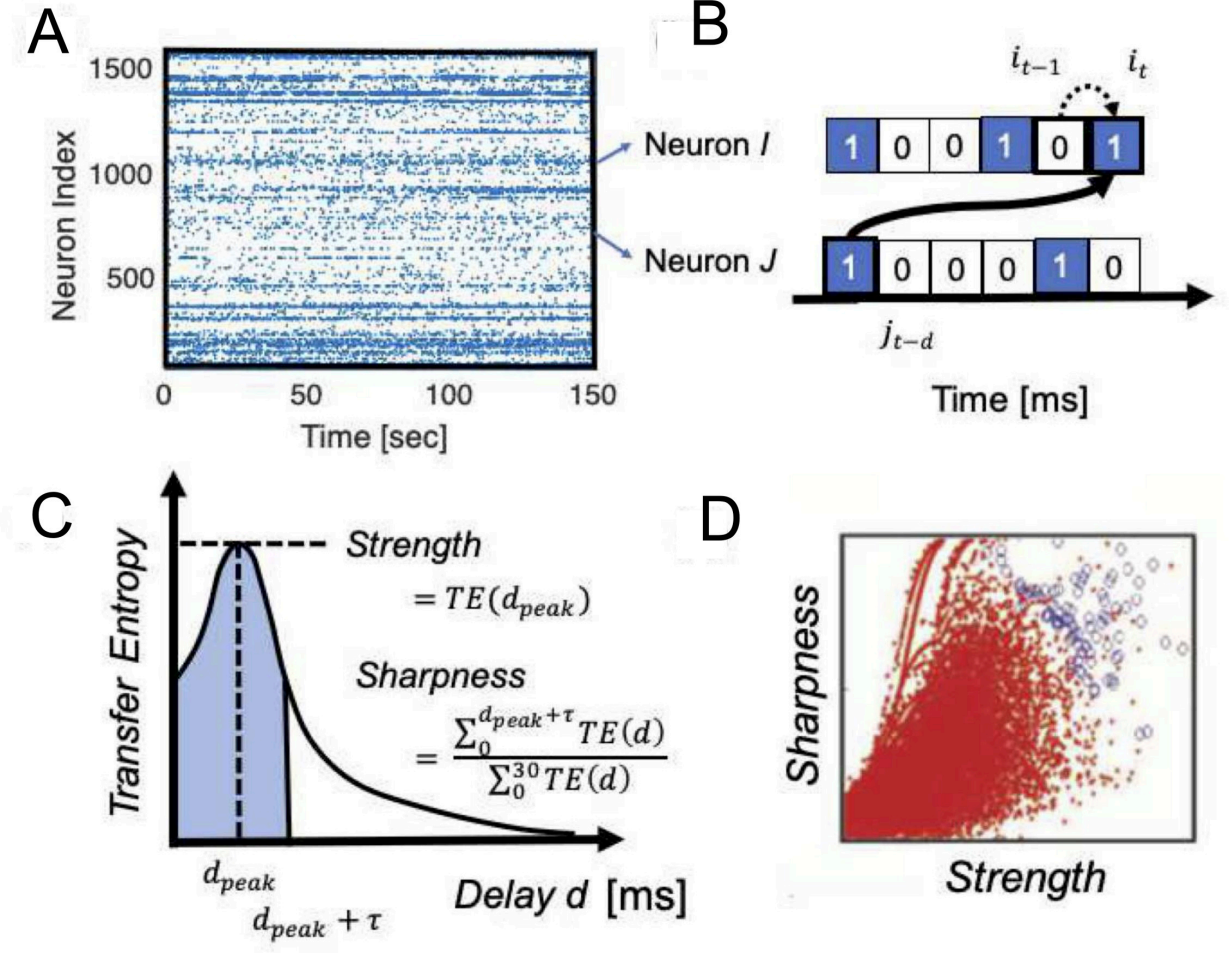
**Effective connectivity definition: A** A raster plot of neuronal spikes given after spike sorting analysis. **B** zoomed-in view of a pair of neurons, I and J. The transfer entropy used in this study specifically quantifies information flow sent from neuron *J* to I that takes delay *d amount of time steps* for the signal transmission, after conditioning on the past activity of neuron *i* at time *t-1*. **C** Computing TE at different delay d, we can get *TE*(*d*), Transfer Entropy as a function of delay d. To characterize the *TE*(*d*) in terms of strength and sharpness of connections, we characterized two variables shown in this panel. τ is 4 ms in this study. The value was selected as the standard deviation of the fitted function to the average of *TE*(*d*) for all neuron pairs. **D** The Strength and Sharpness are mapped onto the two dimensional space and we selected connected pairs of neurons shown as blue circles by comparing with the distribution of shuffled spike data (red dots).

TE is positive if including information about neuron J’s spiking activity improves the prediction of neuron I’s activity beyond the prediction based on neuron I’s past alone (Fig 2A). The equation for TE used in this study is expressed as:

$${TE}_{J\to I}(d)=\sum_{i_{t},\;i_{t-1},j_{t-d}}p(i_{t},i_{t-1},j_{t-d})log(\frac{p(i_{t}|i_{t-1},j_{t-d})}{p(i_{t}|i_{t-1})})$$ (1)

This equation quantifies the expected value of the local transfer entrop*y $log\left(\frac{p(i_{t}|i_{t-1},j_{t-d})}{p(i_{t}|i_{t-1})}\right)$* for all *i*_*t*−1_, *j*_*t−d*_ *and i*_*t*_ [59]. The variables *i* or *j* are given the index of time to express if neuron *I* or *J* is active or not at a time bin. The values are labeled 1 when they are spiking, and 0 when they are not spiking.

The time bin sizes are selected as 1ms, as a common size used to calculate Correlograms or correlations to capture physiologically reliable various phenomana [60–64]. The numerator inside of the logarithm expresses the probability of event *i*_*t*_ in neuron *I* conditioned on its own past *i*_*t*−1_ and the event *j*_*t*−*d*_ of the potentially presynaptic neuron J. The denominator expresses the probability of event *i*_*t*_, but conditioned only on its own past event *i*_*t*−1_.

Schreiber introduced that the difference between these probabilities, as computed by the Kullback-Leibler divergence, which he called Transfer Entropy (1) [59], which gives a quantity that estimates the information transfer from the source variable *J* to the target variable *I*. (Fig 2B). Given that synaptic delays between cortical neurons could span several milliseconds [65, 66], we adopted a version of TE that allows delays between neuron *I* and *J*.

Generally, we can include older past events of *I* and *J* in conditional terms as given in the original definition of TE [59]. This study used only the most recent time bin of neuron *I* and one time bin at delay *d* from neuron *J* (Fig 2B). This is because our past study demonstrated that the connectivity matrix produced from higher-order TE was very similar to ones produced by first order TE, which includes only one time step [16, 38].

The other terms were as defined in Eq 1. TE(d), Transfer Entropy as a function of delay d, between 2 neurons was plotted as a function of different delays *d*, which often showed a distinct peak (Fig 2C). Please note that this delay is essential to determine the directionality of TE since it derives from neuron J’s past activity which serves to predict the future of neuron I. Thus, connections will be directed from past to future.

Additionally, how strong and sharp the peak in the *TE*(*d*) is, becomes essential to the determination of a direct causal influence by synaptic connections existing from neuron J to neuron I [67]. Therefore, we defined the *Strength* as the peak value of *TE*(*d*), and the *Sharpness* as the following equation (Fig 2C):

$$Sharpness=\frac{\sum_{d=0}^{d=d_{peak}+\tau }TE(d)}{\sum_{d=0}^{d=30}TE(d)}$$ (2)

The Sharpness was previously called Coincidence Index [16, 38], and in this paper we use the term *Sharpness* in order to express the intuitive meaning more clearly (Fig 2C). The time window for seeking a peak was 30 ms as the estimated time window for transmitting electrical signal in the spatial size of our recording brain regions [68, 69]. We also used the 30ms to define the denominator of Sharpness, and selected τ = 4 ms, which is the standard deviation for all pairs of neurons, to define the numerator of Sharpness in Eq 2.

Our past studies selected connections having a strong and sharp peak in the TE(d) on the Strength-Sharpness space by comparing the raw connectivity matrix with connectivity matrices generated from 100 shuffled spike sequences. The shuffled spike sequences were prepared by swapping spikes of presynaptic neurons into silent or quiescent time bins within ±10ms (Fig 2D) We selected such connections according to methodologies repeatedly reported in our past studies [16, 38]. We briefly explain the process.

The process consists of five steps: First, we plotted data samples corresponding with neuron pairs in the 2D space of Strength and Sharpness (Fig 2B). Second, we divided the two dimensional space at a resolution of 25×25 pixels. Third, we count how many samples or dots exist about both real data and shuffled data. Fourth, we characterize the ratio of samples between real data and shuffled data existing within individual pixels, we calculated Rejection Threshold (RT) at (*i*, *j*)th pixel on the two dimensional space of *Strength* and *Shapness* according to the following equation:

$$RT(i,j)=\frac{N_{jitt}(i,j)}{N_{real}(i,j)+N_{jitt}(i,j)}(i,j\in [1∼25])$$ (3)

*N*_*real*_ and *N*_*jitt*_ express the number of data samples at (i, j) pixel in the 2D space. Past studies have demonstrated that Strength and/or Sharpness become smaller for shuffled data than real data when originally the Strength and/or Sharpness is strong. this threshold naturally becomes bigger at the top-right region than other regions in the 2D space. Finally, we selected 0.34 as the RT to select connected pairs. The paremter is almost same value as a past study [38]. Refer to the past study in detail.

The shuffled data also gives the baseline to define the bias-adjusted strength of the connection more accurately according to ${IT}_{j\to i}={TE}_{j\to i}^{real}-{TE}_{j\to i}^{shuffle}$. This value is named “strength” in the following manuscript. These connectivities and strengths have been repeatedly evaluated by comparing them with EPSPs measured in patch-clamp recordings [16]. Before connectivity estimation, we categorized neurons into excitatory and inhibitory neurons using the method explained in the following two subsections.

### E/I categorization

As extension of past studies, we additionally calculated a new variable, the sorted local transfer entropy (SLTE). The essence of SLTE is that it sorts the local transfer entropies [36] according to their positive or negative signs for different interactions. Local transfer entropy estimates the information transfer between two events, rather than two variables, so it can quantify how informative the event of a presynaptic spike is to the event of a postsynaptic spike. The following cell categorization process directly utilizes the relationships between pre-synaptic neuron and post-synaptic neurons instead.

SLTE specifically distinguishes between inhibitory influences and excitatory influences, by using a sorting method that takes into account the reversed signs of the local transfer entropies for the excitatory and inhibitory interactions [37].

$${SLTE}_{J\to I}(d)=\sum_{i_{t},i_{t-1},j_{t-d}}p(i_{t},i_{t-1},j_{t-d}){\left(-1\right)}^{\left(i_{t}-j_{t-d}\right)}log(\frac{p(i_{t}|i_{t-1},j_{t-d})}{p(i_{t}|i_{t-1})})$$ (4)

SLTE is similar to the normal Transfer Entropy except that the local transfer entropies are weighted differently, according to the multiplier ${\left(-1\right)}^{\left(i_{t}-j_{t-d}\right)}$. For our case where *i*_*t*_ and *j*_*t-d*_ are 1 or 0, depending on whether the respective neuron’s event is spiking or inactive, this multiplier becomes +1 when both *i*_*t*_ and *j*_*t-d*_ have the same event and -1 when the events are different.

For excitatory interactions, this yields a positive local transfer entropy, whereas for an inhibitory interaction this would yield a negative local transfer entropy. This sorting rule then makes the sum of all terms in SLTE positive for excitatory interactions and negative for inhibitory interactions. Here, an observation of the same events (e.g. a postsynaptic spike at *i*_*t*_ = 1 that follows a presynaptic spike at *j*_*t-d*_ = 1) yields a positive local transfer. Inversely, an observation of unequal events (e.g. a presynaptic spike at *j*_*t-d*_ = 1 not resulting in a postsynaptic spike at *i*_*t*_ = 0) yields a negative local transfer entropy for excitatory interactions. In this quantification, the amount of given information from *p*(*i*_*t*−1_ = 0, *j*_*t−d*_ *= 1)* for rare events *i*_*t*_ = 1 is much bigger than the one from *p*(*i*_*t*−1_ = 1, *j*_*t*−*d*_ *= 1)* for frequent events *i*_*t*_ = 0.

Similarly, for inhibitory interactions the situation is reversed, so that all terms in the sum of SLTE become negative with the sorting rule. Here, observing the same events (e.g. a postsynaptic spike at *i*_*t*_ *= 1* that follows a presynaptic spike at *j*_*t-d*_ *= 1*) yields a negative local transfer entropy, whereas observing unequal events (e.g. a presynaptic spike at *j*_*t-d*_ *= 1* not resulting in a postsynaptic spike at *i*_*t*_ *= 0*) yields a positive local transfer entropy.

**Fig 3:**
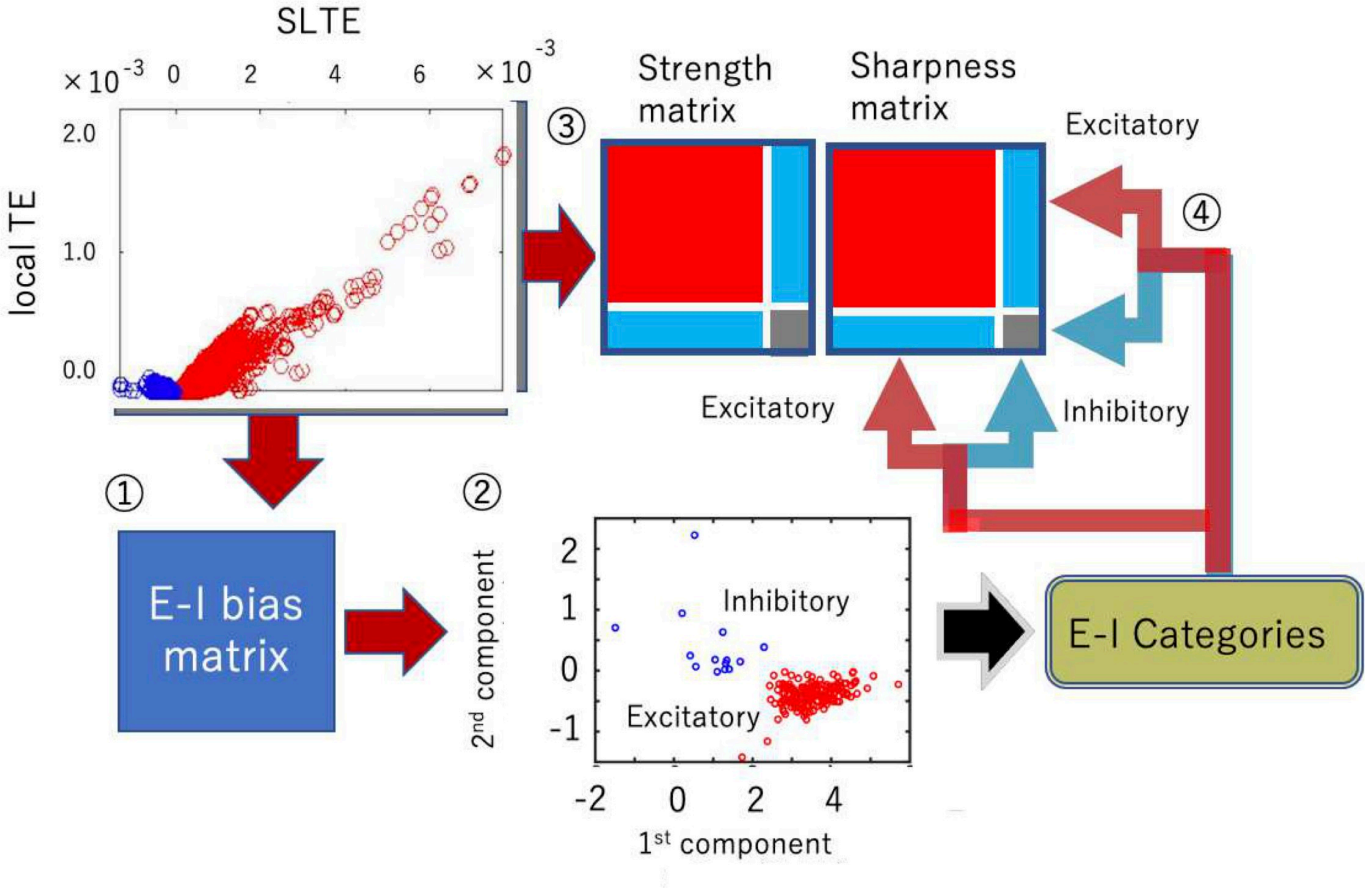
**Four processing steps as a combination of cell categorization and connectivity estimation:** ① First, we calculated E-I bias (SLTE at the delay where TE shows the peak value). ② Then, we determined the sign, positive or negative by observing the clustered distributions in the two-dimensional space of the the 1st component (∝ ∑_*connections in top*90% *about peakTE*_ *SLTE*) and the 2nd SLTEs components (∝ log_10_ *FR*). ③ We calculated Strength and Sharpness from the *TE*(*d*), Transfer Entropy as a function of delay d, according to the analysis scheme shown in Fig 2C and 2D. ④ Finally, we separated the Strength and Sharpness matrices into excitatory and inhibitory groups based on the classification of step ②. (b) The cell categorization preceded the connectivity determination because we should prepare the shuffled null data separately for excitatory and inhibitory synaptic connections to determine connected neuron pairs [Refer to the supplemental material of Shimono, Beggs, 2014 for more detailed information]. We separately produced the connectivity matrix for excitatory and inhibitory neurons by selecting strong or sharp connections as compared with the null shuffled data.

We selected SLTE value at the time delay *d* when TE shows the peak value (Fig 3, upper left panel) in order to highlight the time window where direct synaptical influences exist. We simply call the peak value of SLTE as *E-I bias* in the following sections. The *E-I bias* will hold the positive/negative sign as it comes from Eq 4. In order to carefully define cell labels of excitatory or inhibitory *neurons* from signs given for individual *connections*, we first limited cortical neurons in comparison with staining image to reduce unexpected noise caused by subcortical neurons.

We next sorted or rearranged SLTEs from bigger to smaller based on the order of peak values of TE normalized with influence of firing rate, *TE*_*nor*_ = *TE*_*peak*_/(*FR*∙*log* (*FR*)+(1−*FR*)∙*log*(1−*FR*)), to purely extract independent factors to simple firing rate, or activity level. Then, we calculated the sum of the remaining SLTEs for output connections of individual neurons, after cutting off the lower 10% of the sorted order, and used it as the first component of the E-I bias value. Here, the averaging process translates E-I labels, originally given for individual output connections, to E-I labels for individual neurons based on Dale’s principle [70]. The reason we eliminated 10% of relatively weaker SLTEs is that we want to keep a higher signal-to-noise ratio in comparison with any noises came from non-physiological factors. But the eliminated ratio is small.

The second component was a logarithm of firing rate (At panel ② in Fig 3, although minus and a scaling value are also multiplied to it, it is not essential.). Then, we plotted among all output connections for all individual neurons in the two dimensional space, and categorized them into excitatory and inhibitory neurons by identifying their associated clusters using hierarchical clustering based on Ward variance minimization algorithm, and gave excitatory labels for neurons participating in the cluster locating at the relatively positive side about sum of SLTE as naturally predicted from Dale’s principle.

Before we applied real data, we evaluated the categorization ability in neuronal computational models [71] including inhibitory STDP [72, 73]. These evaluations showed stably ~95% accuracy in various parameter regions for both excitatory and inhibitory neurons (S6A Fig). Other *in vivo* neuronal spike data was also evaluated and revealed similar prediction performance [74] (S6A Fig). We also checked that we were able to get similar categorization performance only with SLTEs. But, we finally decided to use firing rate as the second axis because the combination seems to provide a slightly better categorization performance than only SLTEs. Here, we need to notice that, the difference of firing rate between excitatory neurons and inhibitory neurons was not completely determined by this selection of axes because the way in which the second axis, firing rate, gave signs for the E/I categorization was completely a data driven issue.

### Combining connectivity estimation and E/I categorization

The methods described in the previous two subsections enabled us to get inhibitory and excitatory labels from the *E-I bias* matrix and the connection patterns from the connectivity *Strength*. Next, we examined how we could systematically combine these two procedures, the connectivity estimation (strengths, connected or not) and sign estimation (positive or negative)?

First, we categorized neurons into excitatory or inhibitory neurons (steps ① and ② in Fig 3), and we applied the given neuron categories to *Strength* and *Sharpness* matrices in the following step ④. Recall that we need to prepare a two-dimensional map of *Strength* and *Sharpness* to define connectivity (refer Fig 2). After completing the neuron categorization (Fig 3), we selected connections based on the method shown in Fig 2 separately for four connection categories, i.e. excitatory-excitatory, excitatory-inhibitory, inhibitory-excitatory, and inhibitory-inhibitory connections.

It is critical to note that cell categorization must be performed before we determine the connectivity, because we have to separately compare the real data with the shuffled null data for excitatory and inhibitory synaptic connections.

### Network analyses

Estimation of the connectivity matrix is not the final destination of neuroscience. The estimation was prepared to effectively untangle the complex web of interactions among huge numbers of neurons. This study specifically evaluates the controlling ability of a relatively smaller number of inhibitory neurons to which have stronger influence in the microconnectome than the relatively larger number of excitatory neurons. So, we quantify the relative importance of individual neurons based on their functional connectivity pattern.

Individual neurons are regarded as nodes which are equally including both excitatory and inhibitory neurons. At the same time, a bundle of synaptic connections connecting a pair of nodes are regarded as links or edges. Notice that in our study, directionality was defined for characteristics of all individual links. Let us explain the basic network properties based on these components. The first step and the most naive evaluation of networks is degree or connectivity strengths, and the following second and third steps are quantifications of influences of individual nodes based on centrality and controlling ability respectively.

**Fig 4:**
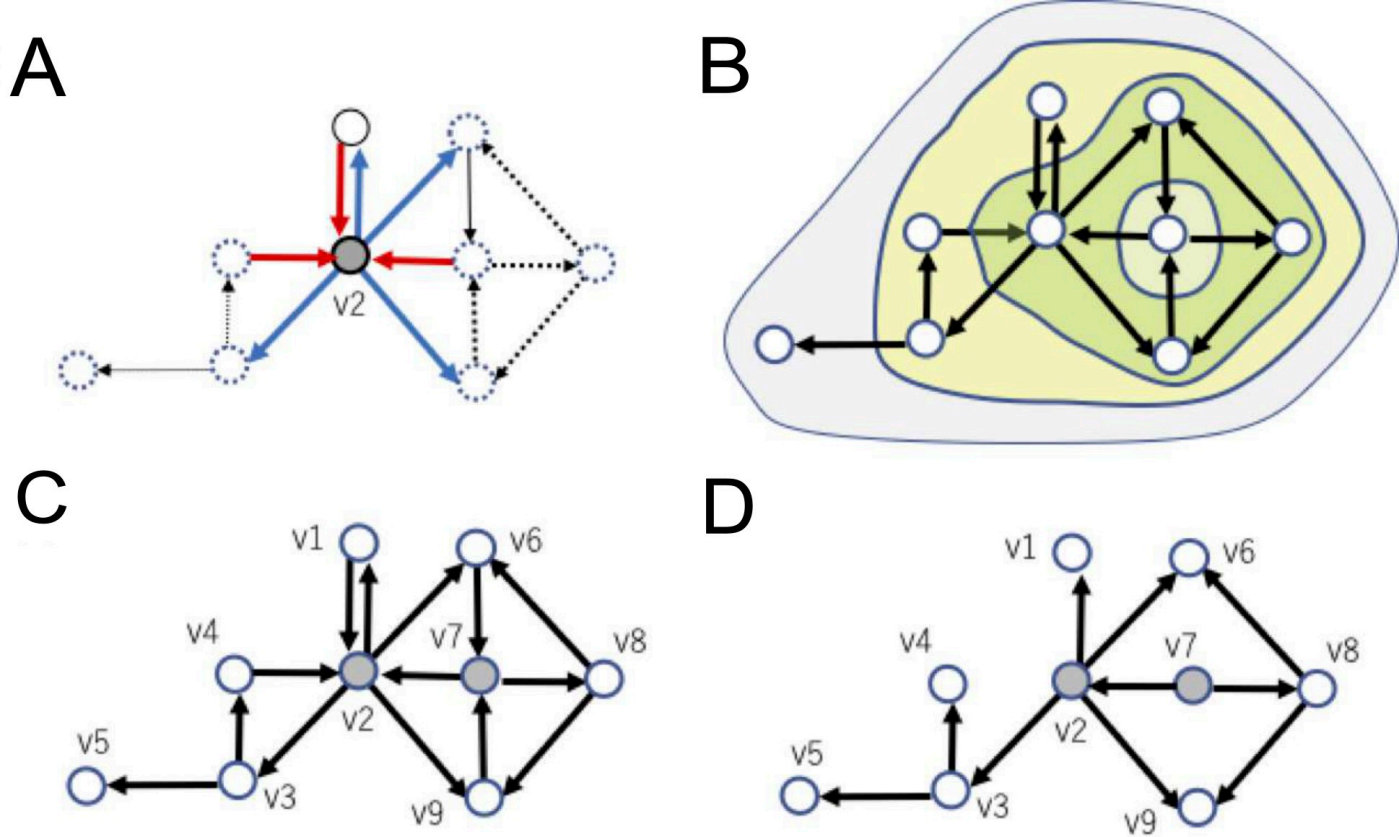
**Example of *network variables* in a *directed connectivity*: A** Out-degree or in-degree expresses how many links connect from or to a specific node respectively, shown as thick lines surrounding node v2 as an example. **B** An example of subsets of nodes extracted with a k-core centrality algorithm. The largest, middle, and smallest regions correspond to 1-core, 2-core, and 3-core, respectively. Note that k’-core is included in k-core for all k’>k. Here, the remaining nodes (e.g. v7) after the final peeling step is also included as the same k-core level that was finally peeled off, such as v2, v6, v8 and v9. **C** Here, an example feedback vertex set (FVS), nodes v2 and v7, are marked with gray color. FVS nodes like this example are defined as a set of nodes. Removal of incoming nodes to them makes the resulting graph acyclic. FVS nodes like this example are defined as a set of nodes after eliminating inputs to the set, the remaining connectivity does not hold directed cycles around the FVS. **D** If we remove incoming links to FVS nodes, all cycles included in the connectivity pattern disappear. This property represents that v2 and v7 are an example of FVS nodes in the system. Furthermore, there is no smaller size FVS. Therefore, {v2, v7} is also an example of MFVS(Minimum Feedback Vertex Set).

The first step and the most naive evaluation of networks is in terms of degree or connectivity strengths, and the following second and third steps are quantifications of influences of individual nodes based on centrality and controlling ability respectively. Degree tells how many connections extend around individual nodes. Degree can be categorized into in-degree and out-degree, which respectively express the numbers of input and output links directly connecting with individual nodes. So, the expression of in-degree and out-degree for a connectivity matrix comprise the total number of input connections and the total number of output connections by regarding matrix as a binary matrix. Histograms of input/output degrees are the key factor to know if there are neurons that have clearly more connections than other nodes (Fig 4A).

The second step of network analysis is evaluation of centralities with k-core centrality. Broadly speaking, degree can be categorized as a kind of centrality measure, where the centrality is a concept for indicating importance of a node. Since there are various criteria and viewpoints to evaluate importance, many centrality measures have been proposed and utilized. In this paper, we use the k-core centrality in addition to the degree centrality because it is known that the k-core centrality better reflects hierarchical properties of networks than the degree and other centrality measures [75]. k-core centrality is defined via k-cores, which are subsets of nodes and are determined in a recursive manner from k = 1 to larger degrees. Precisely, a k-core is obtained by such recursive removal of nodes of degree less than k until all nodes in the remaining graph have degree more than or equal to k (Fig 4B). Note that the k’-core is included in the k-core for all k’ > k. Note also that k-core is not the same as the subset of nodes with degree k or more. For example, all nodes in a *Star*, a connected graph consisting of (n-1) nodes with degree 1 and one node with degree (n-1), belong to the 1-core but not to any k-core with k>1 because the degree of the central node becomes 0 after removing all degree-1 nodes. A node is said to have k-core centrality if it belongs to the k core but not to the (k+1)-core. In this paper, highly connected and highly central nodes will be simply called hubs.

The third step of network analysis is evaluation of Controlling ability through Feedback Vertex Set (FVS). This step provides a viewpoint of *controllability*, more accurately speaking *structural controllability*, to analyze the architecture of effective networks. Here, controllability expresses the ability to guide a system from any initial state to any desired state in a finite time interval by stimulating *driver nodes*. Driver nodes are defined as nodes to which external control inputs are directly given. Specifically, structural controllability focuses on the controllability of a given system characterized only with information of its structural architecture [76]; This approach has been considered superior to discussing whether a system is controllable for almost all parameters without knowing all parameters. This ability has overcome the common issue that traditional control theory has often tried and failed to identify all the parameters (e.g., coefficients). Liu et al. (2011) introduced the concept of *structural controllability* into the research field of complex networks, and many relating questions have been asked in applications of *structural controllability* for network systems; One key question is how to select the minimum number of *driver nodes* to structurally control the whole system as it is realistically difficult that we simultaneously control a huge number of nodes by direct inputs to all of them [77].

There exist three major graph theoretical approaches based on the following characteristics: (i) maximum matching (MM) [28, 77], (ii) minimum dominating set (MDS) [78], and (iii) *feedback vertex set* (FVS) [25, 42]. Each model has some limitations. For example, controlling the targets for MM is limited to linear systems. MDS usually requires several or higher degree nodes to control the system using only a small number of driver nodes. The target states of FVS should be (statically or periodically) stable. The hidden dynamics of neurons behind the connectivity matrix show strong non-linearity, and also do not have many high degree nodes because long-tailed distribution could be observed. Furthermore, the target states are usually considered as stable states because cells in transient states will move to other states soon. Therefore, an FVS-based approach was adopted.

Recalling the definition of FVS, a subset of nodes in a directed graph is called an FVS if removal of input links to nodes in the subset makes the graph acyclic, where acyclic means that there is no directed cycle (i.e., no directed loop). Looking at the example shown in Fig 4C, and imagining the situation when we cut all links incoming to nodes v2 and v7 from the original connectivity pattern (Fig 4D). Then, you will realize that the remaining connectivity will lose all *directed cycles*, *which* are closed loops or cycles created by connecting directed links in series (Fig 4D). Therefore, {v2, v7} is an FVS.

From Fig 4D, it is also seen that all other nodes are located “downstream” of {v2, v7}. This relative position of an FVS implies that, when we control the value of each node in an FVS to be constant, the dynamics of the other nodes will also become sequentially entrained into one stable state after passing a long enough time interval. This property suggests that an FVS can be successfully used as a *driver node set* if the desired state is a steady state. Furthermore, by giving periodically-changing values to nodes in an FVS, we will be able to control the whole system into a set of periodic states. So, FVS nodes can play the role of a *driver node set* when the target states are statically or periodically stable states (i.e., the target states are attractors).

Remember that the current objective is to seek the minimum combination of driver nodes. So, we specifically want to seek the smallest FVS nodes, consisting of the minimum number of nodes, which is called *minimum feedback vertex set* (MFVS) nodes. Since we would generally not try to drive a system to non-steady states, it is a realistic demand that we seek an MFVS, as a minimum size driver node set. The FVS-based approach can be also applied to wider classes of non-linear systems as well as linear systems [25]. Furthermore, the FVS-based approach was applied to the analyses of gene regulatory networks [25, 27] and signaling pathways [26]. The results suggest that nodes in MFVS or nearly minimum feedback sets often correspond to genes or proteins playing important biological roles.

It is important to note here that MFVS is not necessarily unique; For example, the set of {v2,v8} is also an MFVS. So, we would be better to categorize nodes that are part of MFVS nodes into two groups, one group is nodes participating in all MFVS nodes [26], and another group is nodes participating in one of the MFVS nodes but not in all of them. These are called *critical* and *intermittent* nodes respectively, and the remaining nodes are called redundant nodes [79]. In the example shown in Fig 4D, v2 is the only one critical node v7, v8 are intermittent nodes, and others are redundant nodes. Although this report does not treat differences between intermittent FVS nodes, and others are redundant nodes in order to simplify, the difference will be also examined in future studies.

Currently, it is known that the computation of an MFVS is NP-hard. This implies that it is not plausible that there exist theoretically efficient algorithms for finding an MFVS. It is possible in practice to compute an MFVS for up to moderate size networks using Integer Linear Programming (ILP). We employed this simple ILP formalization to compute MFVS nodes and types of nodes, using IBM ILOG CPLEX as a solver for ILP.

## Results

### Basic properties

**Fig 5:**
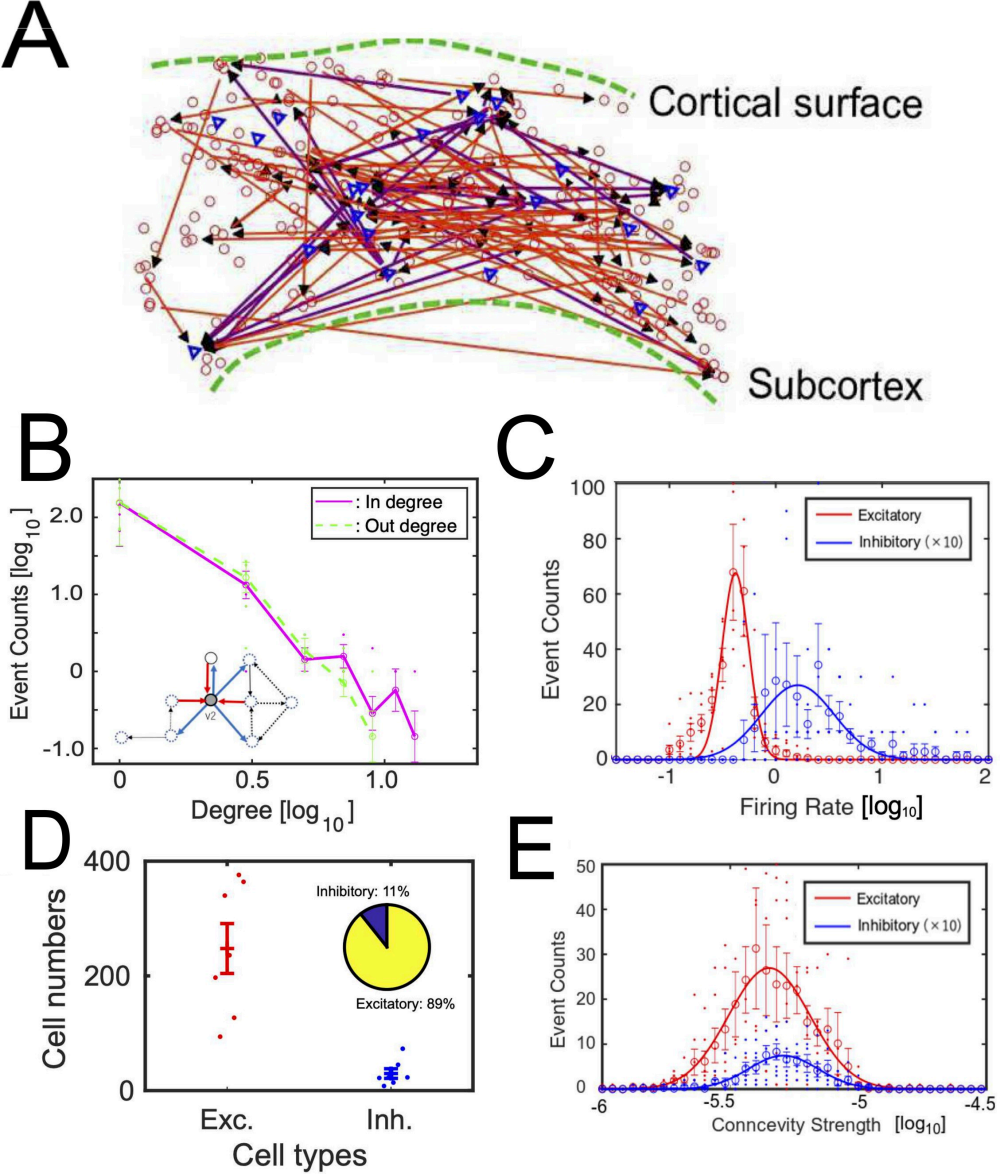
**Basic properties of neuronal networks: A** shows an example of iInteraction networks among neurons. Triangles are inhibitory neurons and circles are excitatory neurons. The upper side corresponds to the cortical surface. **B** shows degree histograms, histograms of number of connections. The solid line and dotted line show results for out degree and in degree for all neurons respectively. Small inserted figure shows input connections to and output connections from node v2 in the same example shown in Fig 4. Refer to degree histograms only for excitatory or inhibitory neurons at S2 Fig. **C** shows histograms of firing rate for excitatory neurons (solid line) and inhibitory neurons (dotted line). Because the number of inhibitory neurons is much smaller than the number of excitatory neurons, we multiplied 10 to the histogram of inhibitory neurons. **D** shows the numbers of identified excitatory and inhibitory neurons for seven cortical slices. The inserted pie chart shows the relative ratio of their numbers. The confidence intervals on the ratio of inhibitory to excitatory cells was 0.07–0.18. **E** shows histograms of connection strengths for excitatory neurons (solid line) and inhibitory neurons (dotted line). The meaning of is the same as the panel C. The x-axis of panels B, C and E are log scale with base 10. Error bars in panels B-E express their standard errors.

We found ~1000 neurons by applying spike sorting to electrical activities of 7 acute brain slices recorded over 2.5 hours at somato-motor cortex of mice (n = 7, aged 3–5 weeks). We carefully extracted cortical regions and defined borders between different cortical layers based on immunohistochemistry staining data. The following network estimation method provided complex networks in the extracted cortical region as exemplified in Fig 5A. The relative ratio of inhibitory neurons was found up to 15%. The ability of E/I categorization methods are also validated in in vivo spike data and a computational model (S6 Fig).

It is well known that direct observations of such examples do not simply enable us to capture the detailed topology. Therefore, first, we started by quantitative observation of the histograms of degrees, number of connections, for individual neurons of the excitatory and inhibitory cases from reconstructed effective networks among neurons (Fig 5B). In-degree is the number of input connections and out-degree is the number of output connections. For example, both in-degree and out-degree at node v2, shown as the small insert in Fig 5B, are 3. Fig 5B shows the histogram of in-degree and out-degree on a log-log space. The result shows that the degree histogram follows a long-tailed form and that there are hubs and nodes having exponentially more numbers of connections than others, not only for excitatory neurons but also for inhibitory neurons (Fig 5B). The existence of hubs for excitatory effective connectivity had been already reported in past studies [16, 20]. Here, because the number of samples on the right tail of the distribution is still small, we will need more data to discuss, for example, if the long-tailed distribution is scale-free or not.

Next, we started to observe the rules of the activities of excitatory and inhibitory neurons. Their firing rates approximately followed log-normal distributions (Anderson-Darling test, p<0.05, AD = 0.74). Although the log-normal distribution of the firing rate among excitatory neurons is a relatively well-known basic property, the log-normal rule of firing rate among inhibitory neurons is not well-established yet. As a similar aspect in terms of log-normal rule, we observed the distributions of connectivity strengths of effective networks [16, 38]. This study demonstrated that the connectivity strengths not only among excitatory connections but also among inhibitory connections follow a log-normal rule (Fig 5E; Anderson-Darling test, p<0.05, AD = 0.83). These trends are robustly observed for different network densities. One example is shown at S3 Fig. Notice that we meet with exceptionally stronger connections in a small number of neurons in log-normal distributions than normal distributions. Therefore, contributions within the core system in log-normal distributions is more important than ones in normal distributions. The relative ratio of numbers of excitatory neurons was around 90% (atio of excitatory neurons: 0.89 (mean) +/- 0.12 (90% confidence level); Fig 5D). Now, from the following subsections, we will start to check if inhibitory neurons locate more “influential” relative positions in the network architecture than excitatory neurons from the following subsections.

### Estimations of influential neurons based on centrality

The first perspective to quantify *influence* of individual neurons is how central the positions of the individual neurons are. To characterize such *centrality* of individual neurons, we adopted *k-core centrality*. Here, k-core means the remaining nodes after recursively removing nodes of degree less than k. Nodes participating in a (k+1)-core, but which are removed when removing nodes of degree k, are labeled to have (k+1)-core value. The k-core value will quantify a *centrality*, indicating how central positions individual nodes are located at.

**Fig 6:**
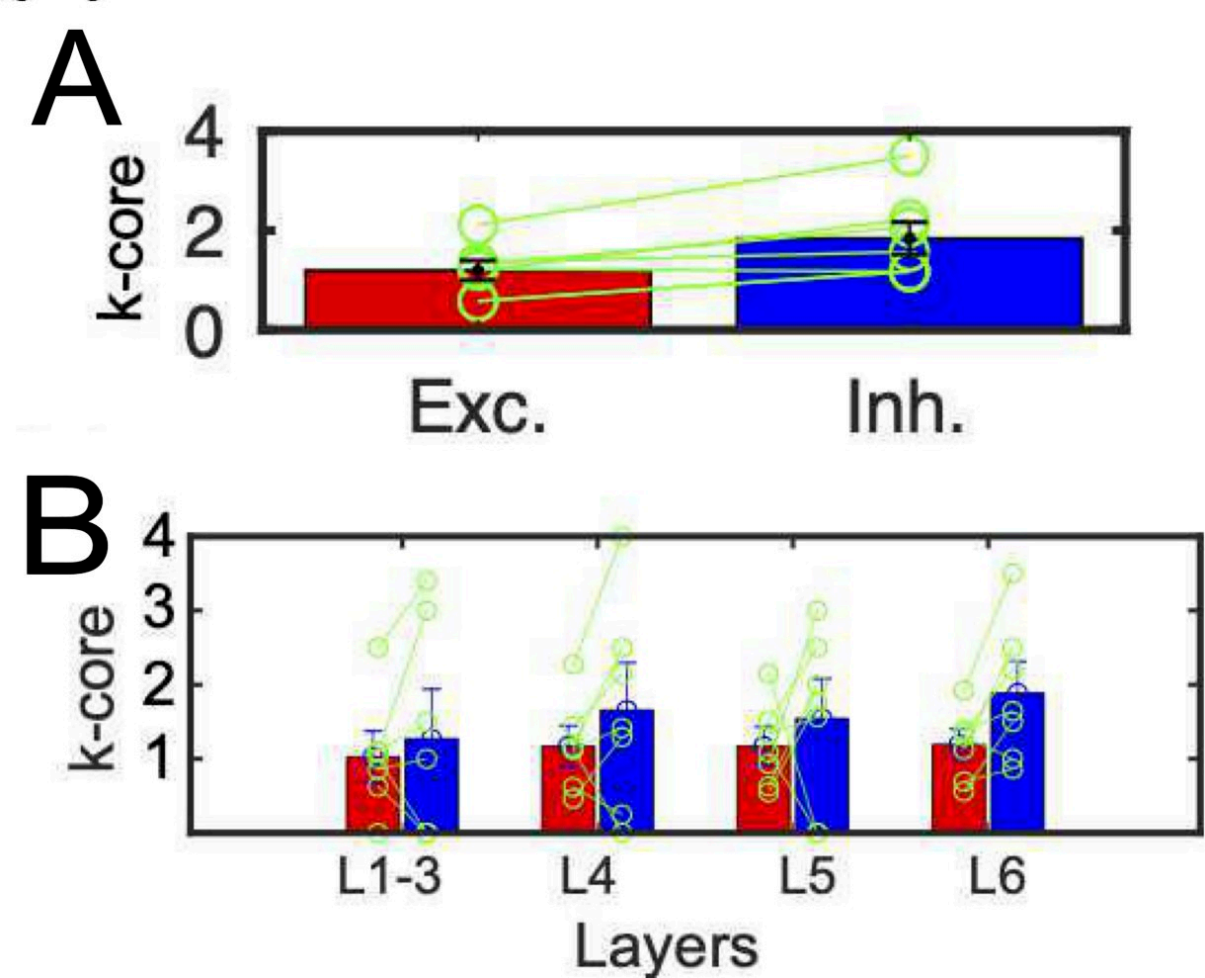
**E/I cell categories and k-core centralities: A** shows the difference of averaged k-core values for all excitatory and inhibitory neurons. The error bars and circles correspond with the variety among 7 cortical slices. They showed significant differences (p<0.05, Wilcoxon signed rank test, means and variances of k-core centrality for excitatory neurons and inhibitory neurons: 1.20±0.27 vs. 1.87±0.72; Z (7 slices) = 2.45). This trend was also observed even for only connected components. **B** shows the difference of averaged k-core values for excitatory and inhibitory neurons within individual layers. The left and right bars for individual layers correspond with excitatory and inhibitory neurons respectively. We performed a non-parametric paired statistical test between excitatory neurons and inhibitory neurons separately for different layers, and shown as * mark if significant difference existed (p<0.05, Wilcoxon signed rank test; layer1-3: 1.02 ± 0.49 (excitatory) vs. 1.27± 1.78 (inhibitory); Z(7 slices) = 0.36, p = 0.34; layer4: excitatory, 1.17 ± 0.30 vs. inhibitory, 1.66 ± 1.62; Z(7 slices) = 0.79, p = 0.15; layer5: excitatory, 1.17 ± 0.26 vs. inhibitory, 1.54 ± 1.15; Z(7 slices) = 0.86, p = 0.29; layer6: excitatory, 1.18 ± 0.18 vs. inhibitory, 1.87 ± 0.64; Z (7 slices) = 2.8, p = 0.04). All error-bars in panels A and B express standard errors.

Through such quantification of centrality, we could observe that the averaged values of k-core centrality of inhibitory neurons are higher than the ones of excitatory neurons for all data samples for all 7 cortical slices, Fig 6A and 6B). This trend could be observed for neurons having at least one connection (S4A Fig), and the ratio of the most central core within inhibitory neurons is also significantly higher than the ratio of the most central neurons within excitatory neurons (S4B Fig). The total ratios of higher k-core node than 1, 2 and 3 were also higher in inhibitory neurons than excitatory neurons (Ratio(k-core> = 1): excitatory, 0.10 ± 0.04 vs. inhibitory, 0.46 ± 0.06; Z (7 slices) = 2.6, p <0.05; Ratio(k-core> = 2): excitatory, 0.10 ± 0.04 vs. inhibitory, 0.38 ± 0.08; Z (7 slices) = 1.7, p <0.05; Ratio(k-core> = 3): excitatory, 0.02 ± 0.002 vs. inhibitory, 0.20 ± 0.07; Z (7 slices) = 1.70, p <0.05, Wilcoxon signed rank test). Also, the superiority of inhibitory neurons in terms of centrality was especially significant in deeper layer 6 because only layer 6 showed significance about k-core centralities (Fig 6B).

### Estimations of influential neurons based on FVS

**Fig 7:**
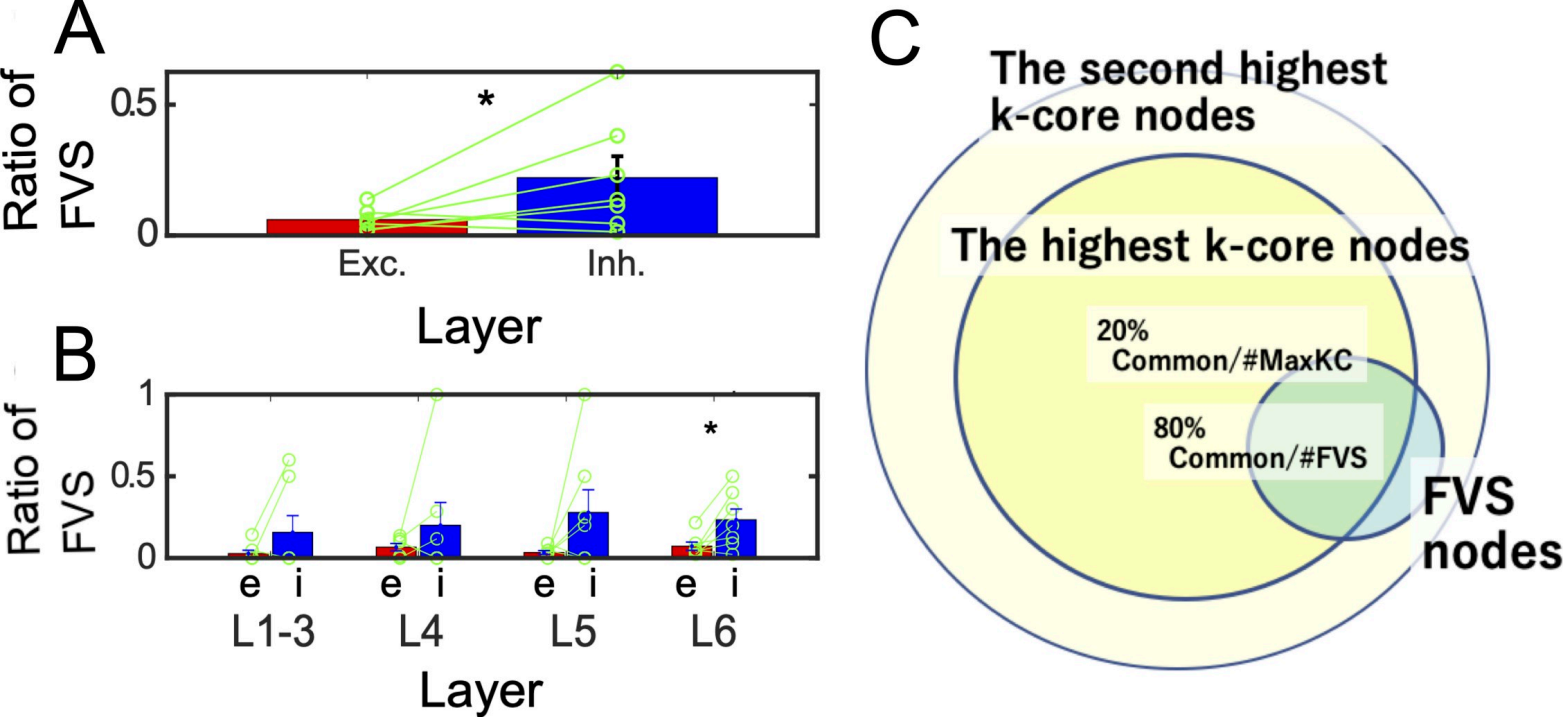
**E/I cell category** **and *FVS (Feedback Vertex Set)*: A** shows the difference of ratios of *FVS* for excitatory (or inhibitory) neurons. Ratios of *FVS* for excitatory (or inhibitory) neurons are defined as *ratios* of number of excitatory (or inhibitory) FVS neurons per the number of all excitatory (or inhibitory) neuron pools. The E/I difference also showed significant difference (p<0.05, Wilcoxon signed rank test (one sided), FVS(excitatory): 0.059±0.001 vs. FVS(inhibitory): 0.226 ± 0.053; Z (7 slices) = 8.3). **B** shows the result of the same statistical tests separately applied to different layers (Wilcoxon signed rank test). We plotted * mark if significant difference existed (p<0.05, Wilcoxon signed rank test (one sided), Layer2-3: FVS(excitatory): 0.027±0.003 vs. FVS(inhibitory): 0.16 ± 0.07; Z (7 slices) = 4.7, p = 0.5; Layer4: FVS(excitatory): 0.067 ± 0.004 vs. FVS(inhibitory): 0.20 ± 0.14; Z (7 slices) = 2.6, p = 0.44; Layer5: FVS(excitatory): 0.034 ± 0.001 vs. FVS(inhibitory): 0.28 ± 0.13; Z (7 slices) = 4.8, p <0.16; Layer6: FVS(excitatory): 0.069 ± 0.003 vs. FVS(inhibitory): 0.26 ± 0.05; Z (7 slices) = 10.1, p <0.05). Here, while Layers 1 to 5 didn’t have any significant difference between the *FVS ratios* for ratio of *FVS nodes* among excitatory and *FVS ratios* for inhibitory neurons, we could find significantly more inhibitory FVS nodes in Layer 6. All *error-bars* in panels A and B express standard errors. **C** The Venn diagram expresses how FVS nodes and nodes having highest *k-core value* overlap each other. About 80% of *FVS nodes* have the highest *k-core value*, and about 20% of nodes having the highest k-core value are categorized as *FVS nodes*. All FVS nodes were included in the group of nodes having the k-core values more than the second highest value.

The second perspective to quantify “*influence*” of individual neurons is about their abilities to control other neurons. We selected nodes having high controlling ability as minimum FVS (Feedback Vertex Set), and As the result of evaluation of we defined Ratio of FVS for excitatory (or inhibitory) as ratio of the number of excitatory or inhibitory FVS neurons per the total number of excitatory (or inhibitory) neurons. As the result of evaluation of Ratio of FVS, we could find the relative influence of inhibitory neurons is significantly stronger than excitatory neurons (Fig 7A). Additionally, we also observed the FVS values between excitatory and inhibitory neurons separately among different layers (Fig 7B). We could also find the superiority of inhibitory neurons is significant at deep layers (layers 6). Notice that inhibitory neurons mainly project to excitatory neurons, so the controlling targets are basically excitatory neurons (100×*Degree*(*i*→*e*)/(*Degree*(*i*→*e*)+*Degree*(*i*→*i*)) = 91.9±0.16[%]).

FVS nodes, which were selected as a group of nodes having high controlling ability of other nodes, are relatively more keenly limited than the higher k-core nodes. Actually, if we observe the Venn diagram between FVS nodes and nodes having maximum k-core centrality value, we could observe FVS nodes are selected as a much more limited group than the highest k-core (FVS common/FVS: 76.4± 32.0 [%] vs. FVS common/KC max: 19.9± 0.17 [%]; Z (7 slices) = 53.4, p <0.05, Fig 7C). Supplementarily, we also could find all FVS neurons are included at least at the second highest k-core groups. Additionally, although we tried to characterize FVS neurons and inhibitory neurons in comparison with local representation, quantified with motifs of three nodes, we could not find any significant trends, such as that FVS neurons more often than non-FVS neurons at highly clustered motifs or that inhibitory neurons also join in the highly clustered motifs more often than excitatory neurons (S5 Fig).

## Discussion

### Short summary

The brain is a non-random network system. We sought to answer how inhibitory neurons are able to keep the balance against excitatory neurons, which are more abundant than inhibitory neurons by seeing the neuronal network as a non-random network system. This study has tried to move forward to reveal how the stronger influence of inhibitory neurons is supported within the network topology of functional interactions among ~1000 neurons beyond simple differences such as among firing rates.

### Log-normal rules

First, we started to observe the difference of firing rate and connectivity strengths of effective connectivity between excitatory and inhibitory neurons. These parameters were previously observed only for excitatory neurons [16, 38]. In this study, we tried to add more information by separately observing excitatory and inhibitory neurons. We could observe that the firing rate in inhibitory neurons is higher than excitatory neurons, as known before. Additionally, we could commonly observe log-normal like distributions, although it cannot be said for certain that the fitting is perfect, for connectivity strengths and firing rate of inhibitory neurons. Past computational simulation study demonstrated that a Hebbian learning rule can produce the situation where both firing rate and connectivity intensity show log-normal distributions [80]. However, the experimental tests for inhibitory neurons have still been rare. A simple mechanism causing the phenomenon that effective connectivities obey log-normal distribution might be that the underlying "structural" connectivity strengths, reflecting the spine sizes obey a multiplicative rule [81]. The relationships between strengths of effective connectivity and spine sizes will be necessary to be checked in future studies, and how much percentage of synaptic connections may effectively work in usual information processes.

In summary, the finding that inhibitory neurons obey log-normal rule hasn’t been well-established. Our finding is new and provides several suggestions. Now, we started to ask more questions about the relative positions of E/I neurons in the network topology and also their controlling abilities of others.

### Influence of neurons in terms of centrality and controlling ability

When we observe the histograms of link numbers of individual neurons, which means degree histograms, we see that they are long-tailed. From this point, past study demonstrated that there are hubs among excitatory neurons [16, 82], and rich club organization [38, 83, 84]. This study quantified the relative difference of influence levels between inhibitory and excitatory neurons according to how central these neurons are located. We could verify that inhibitory neurons are relatively more influential than excitatory neurons in the topological architecture. Additionally, we could find that the significantly more central property of inhibitory neurons than excitatory neurons could be observed in deep cortical layers by combinational evaluations of the effective networks with NeuN immunochemistry staining.

Furthermore, we also tried to extract the most “influential” neurons in terms of their controlling ability of other neurons as the minimum combination of Feedback Vertecs. Then, we could also observe the stronger controlling ability of inhibitory neurons compared to excitatory neurons, and interestingly, again the influential inhibitory neurons are selectively located in deep cortical layers.

Because high centrality and high controlling ability are not mathematically directly related to each other, these two findings infer the high “influence” of inhibitory neurons affect many different aspects of functionality of the neural system. So, briefly speaking, the results suggest that inhibitory neurons play a role of a central controlling regulator under external stimulation and learning processes [85]. We hope such idea about regulation of inhibitory neurons of other neurons in the complex neuronal system will also help to clarify how animal behaviors are built by considering external inputs to focusing brain regions [86]. Because our current experimental data was not bursty, simpler clustering algorithms might be also available to select effectively connected pairs.

### What “influential” inhibitory neurons in deep cortical layers are?

If we limit studies reporting about individual layers, we are able to find consistent results in past morphological studies with our findings: For example, Frandolig et al. reported that parvalbumin inhibitory interneurons in 6a layer of somatomotor cortex locate at the position where is relatively easier to integrate local and long-distance inputs [87]. Additionally, several studies have reported inhibitory interneurons in layer 5 of somatomotor and visual cortex play key roles to control cortical output signals [88, 89]. Furthermore, Kawaguchi et al. reported inhibitory fast spike neuron in layer 5 of frontal cortex has more connections than excitatory neurons [90, 91].

We predict that inhibitory neurons, showing high degree and high controlling ability, may correspond with neurons identified in the past studies. At same time, notice that it has also not been an easy task that we extract such special relative position of inhibitory neurons in deep layers from recording within only individual layers. Although there are many consistent properties, it is necessary to notice that cell types defined with functional properties do not always correspond with cell types defined by morphologies. Therefore, to capture a unified view, further studies about morphological type is also necessary in the future.

It might be said that the connectivity density is low. We suppose that the one reason for the low density might be because our recording device recorded data approximately in two-dimensional space. The solid angle given from flat brain blocks should be much smaller than ones given from a fully three-dimensional recording. Therefore, the connecting targets are limited, and the connectivity density decreases. However, this study carefully produces slices as diagonal angle against the cortical surfaces. Therefore, the connectivity pattern on the recording surface would be well-captured, and it will characterize interactions in individual columnar structures existing on the surface of slices. Especially, we should also need to notice that relatively small numbers of stronger nodes have exponentially stronger influence than other small connections in a topology like lognormal and scale-free like architecture. Therefore, network architecture among strong connections will provide stable results about core architecture of neuronal networks. However, notice that, for example, when we talk about exponentially weaker connections, more careful discussion including real experimental noises will be necessary. Widely speaking, new advances in recording technology to record neuronal electrical spikes in *ms* time resolution from *wide* spatial range covering all cortical layers in the *three-dimensional* space simultaneously will be also awaited. Sub-millisecond resolution information will also potentially help to achieve better analyses schemes. About *C*.*elegans*, molecular mechanisms underlying E/I balance are also becoming clearer than before [92]. Comparisons with molecular-level information with holding spatial maps may also become more important in the future.

## Final remarks

The Brain consists of a huge number of components and neurons. Their connections are intertwined with each other in a very complex manner. In order to understand the system properties, we need to unwind the complex connectivity. This study asked how inhibitory neurons play different roles than excitatory neurons and keep balance with them, and we found the specific characteristics of inhibitory neurons in terms of centrality and controlling ability of other neurons. We also find that such highly central and controller inhibitory neurons are selectively located at deep layers. This statement is very simple, but it was achieved by utilizing cutting-edge recording technology, and sophisticated accumulations of analysis methods. As mentioned in the introduction, E-I balance is still an important basis to interpret many brain diseases. Topological view of many excitatory and inhibitory neurons, demonstrated in this study, will help to move one step forward to interpret such disease, for example, through more detailed data analyses and more realistic computational modeling than was ever possible before.

## Supporting information

S1 Fig Three dimensional expression of recorded brain regions. Black surfaces express the positions of recording brain slices, and we placed a Multi Electrode array at the square regions surrounded by yellow lines. In order to clarify the anatomical names, we overlapped the slice locations with Allen mouse brain atlas (https://mouse.brain-map.org/)). The light blue area on the cortical surface expresses the primary motor region, and the light green expresses primary somatosensory region. We can find the recorded region (yellow square) locates within the primary motor or primary sensory region.
(TIFF)

S2 Fig Four panels express degree histograms only for connections from excitatory neurons to excitatory neurons **A**, from-excitatory-to-inhibitory neurons **B**, from-inhibitory-to-excitatory neurons **C**, and from-inhibitory-to-inhibitory neurons **D**. In all panels, solid lines express in-degree histograms, and dotted lines express out-degree ones. Error bars are standard errors for seven slices.
(TIFF)

S3 Fig Basic topologies for a higher density connectivity map (the rejection threshold is 0.40): **A** shows in-degree or out-degree histograms for all neurons, and **B** shows histograms of connectivity strength. Error bars are standard errors for seven slices.
(TIFF)

S4 Fig Referential figures of comparing k-core values between excitatory and inhibitory neurons. **A** is a very similar evaluation of k-cores with Fig 6A, but the centralities are evaluated only from neurons having at least one connection (degree > 0). Then, we could again observe significant differences between excitatory and inhibitory neurons again (Wilcoxon signed rank test; p<0.05, means and variances for excitatory neurons and inhibitory neurons: 1.47±0.16 vs. 1.98±0.64; Z (7 slices) = 2.11). **B** shows the ratio of neurons having the highest k-core value for individual slices. The values of inhibitory neurons are significantly higher than ones of excitatory neurons (Wilcoxon signed rank test; p<0.05, ratio for excitatory, 0.18 ± 0.03 vs. ratio for inhibitory, 0.40 ± 0.05; Z (7 slices) = 2.7).
(TIFF)

S5 Fig Comparisons with motif histogram. A comparison between how much more often triangle motifs could be significantly observed when comparing with expected numbers, which were calculated from the probability of non-connected or directed or bidirectional connections for pairs of nodes, for FVS of non-FVS neurons (upper panel) and excitatory and inhibitory neurons (lower panel). The connectivity density becomes higher from left to right. We could not find significant trend that FVS and inhibitory neurons have more opportunity to join in more clustered motifs (Wilcoxon signed rank test; p>0.05, FVS(motif> = 8), 9.91×10^7^±2.54×10^8^ vs. non−FVS(motif> = 8), 9.04×10^6^±1.54×10^7^; Z (7 slices)>>10), Inhibitory (motif> = 8), 4.13×10^7^±8.98×10^7^ vs. excitatory (motif> = 8), 6.69×10^6^±1.76×10^8^; Z (7 slices)>>10). The motifs were calculated using Brain Connectivity toolbox [93].
(TIFF)

S6 Fig Evaluation of accuracy about categorization of inhibitory neurons. **A** Evaluation of inhibitory neurons in dataset recorded at rat cortex. The dataset consists of six data sets (2 probes are recorded from 3 rats). The pairs of probes are V1 and PPC, V1 and M2, M2 and PPC. We analyzed all probes independently, and compared the predicted inhibitory neurons and fast spike neurons estimated in the reference [74]. The final result is shown as median value among six data set. Refer the report in more detail about the utilized spike data. **B** Similar evaluation of inhibitory neurons estimated by our methods with answers in a computational model (https://github.com/Motoki878/model). Although we did not show here, prediction of excitatory neurons also showed 99% accuracy.
(TIFF)
