# Supplementary figures and images for: Inhibitory neurons exhibit high controlling ability in the cortical microconnectome
Source: PLoS Comput Biol. 2021 Apr 8;17(4):e1008846. doi: 10.1371/journal.pcbi.1008846 (PMC8031186; doi:10.1371/journal.pcbi.1008846)

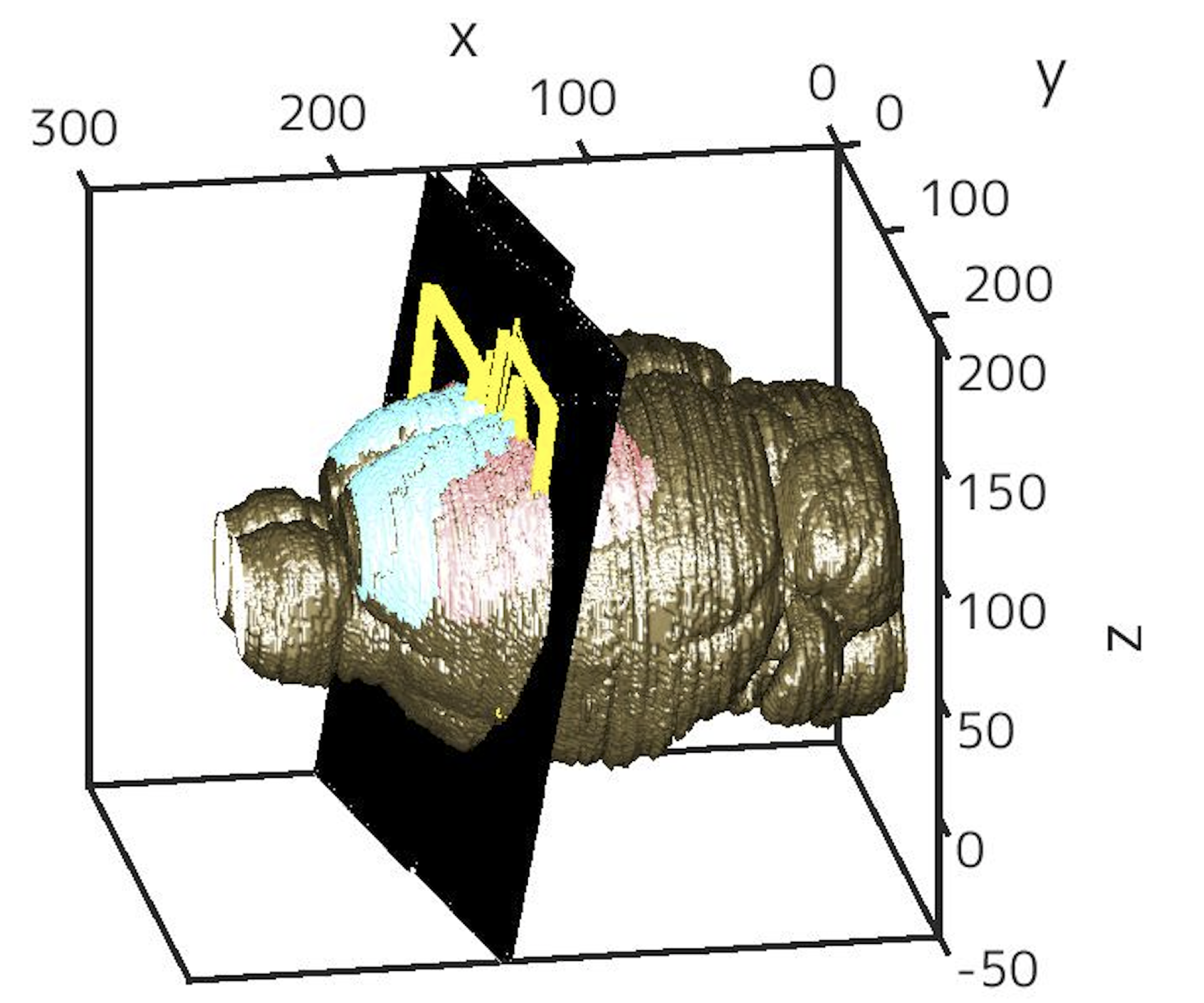

Supplement: S1 Fig — Black surfaces express the positions of recording brain slices, and we placed a Multi Electrode array at the square regions surrounded by yellow lines. In order to clarify the anatomical names, we overlapped the slice locations with Allen mouse brain atlas (https://mouse.brain-map.org/)). The light blue area on the cortical surface expresses the primary motor region, and the light green expresses primary somatosensory region. We can find the recorded region (yellow square) locates within the primary motor or primary sensory region. (TIFF) [file pcbi.1008846.s001.tiff]

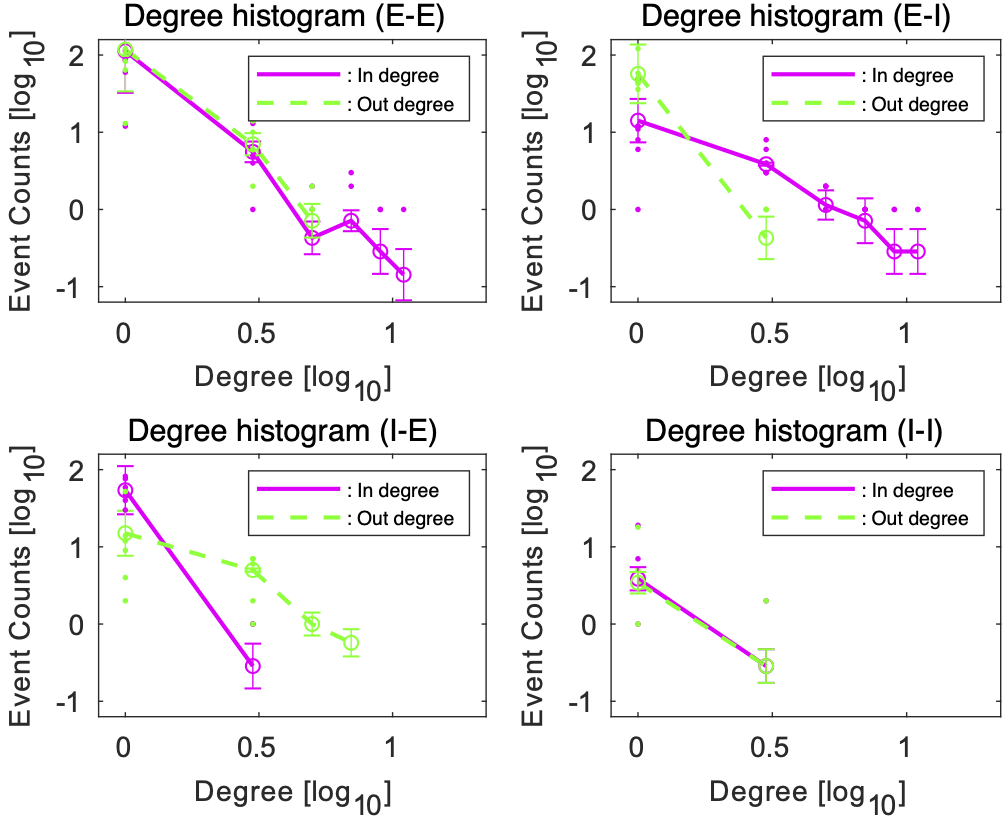

Supplement: S2 Fig — Four panels express degree histograms only for connections from excitatory neurons to excitatory neurons A, from-excitatory-to-inhibitory neurons B, from-inhibitory-to-excitatory neurons C, and from-inhibitory-to-inhibitory neurons D. In all panels, solid lines express in-degree histograms, and dotted lines express out-degree ones. Error bars are standard errors for seven slices. (TIFF) [file pcbi.1008846.s002.tiff]

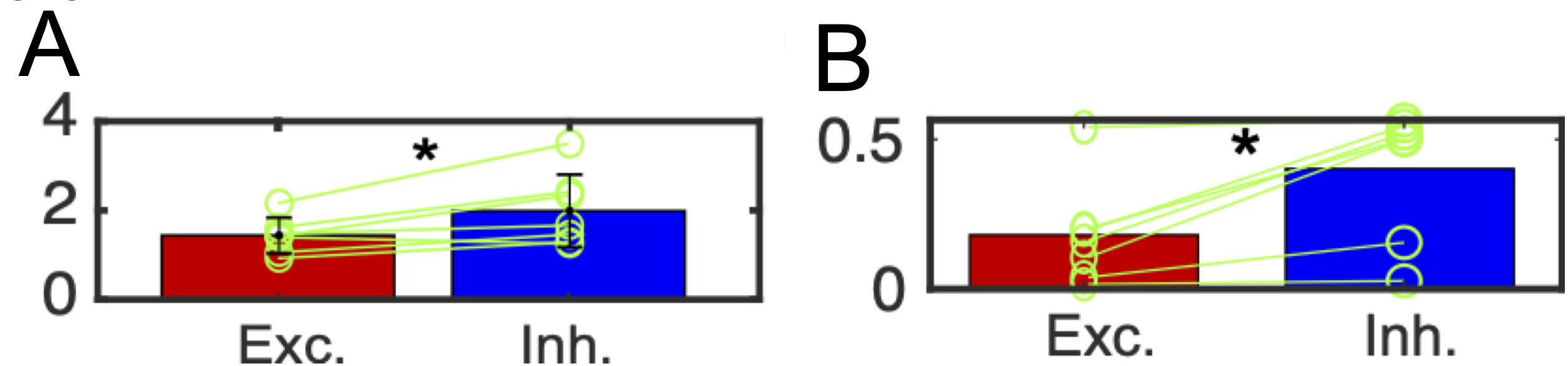

Supplement: S4 Fig — Referential figures of comparing k-core values between excitatory and inhibitory neurons. A is a very similar evaluation of k-cores with Fig 6A, but the centralities are evaluated only from neurons having at least one connection (degree > 0). Then, we could again observe significant differences between excitatory and inhibitory neurons again (Wilcoxon signed rank test; p<0.05, means and variances for excitatory neurons and inhibitory neurons: 1.47±0.16 vs. 1.98±0.64; Z (7 slices) = 2.11). B shows the ratio of neurons having the highest k-core value for individual slices. The values of inhibitory neurons are significantly higher than ones of excitatory neurons (Wilcoxon signed rank test; p<0.05, ratio for excitatory, 0.18 ± 0.03 vs. ratio for inhibitory, 0.40 ± 0.05; Z (7 slices) = 2.7). (TIFF) [file pcbi.1008846.s004.tiff]

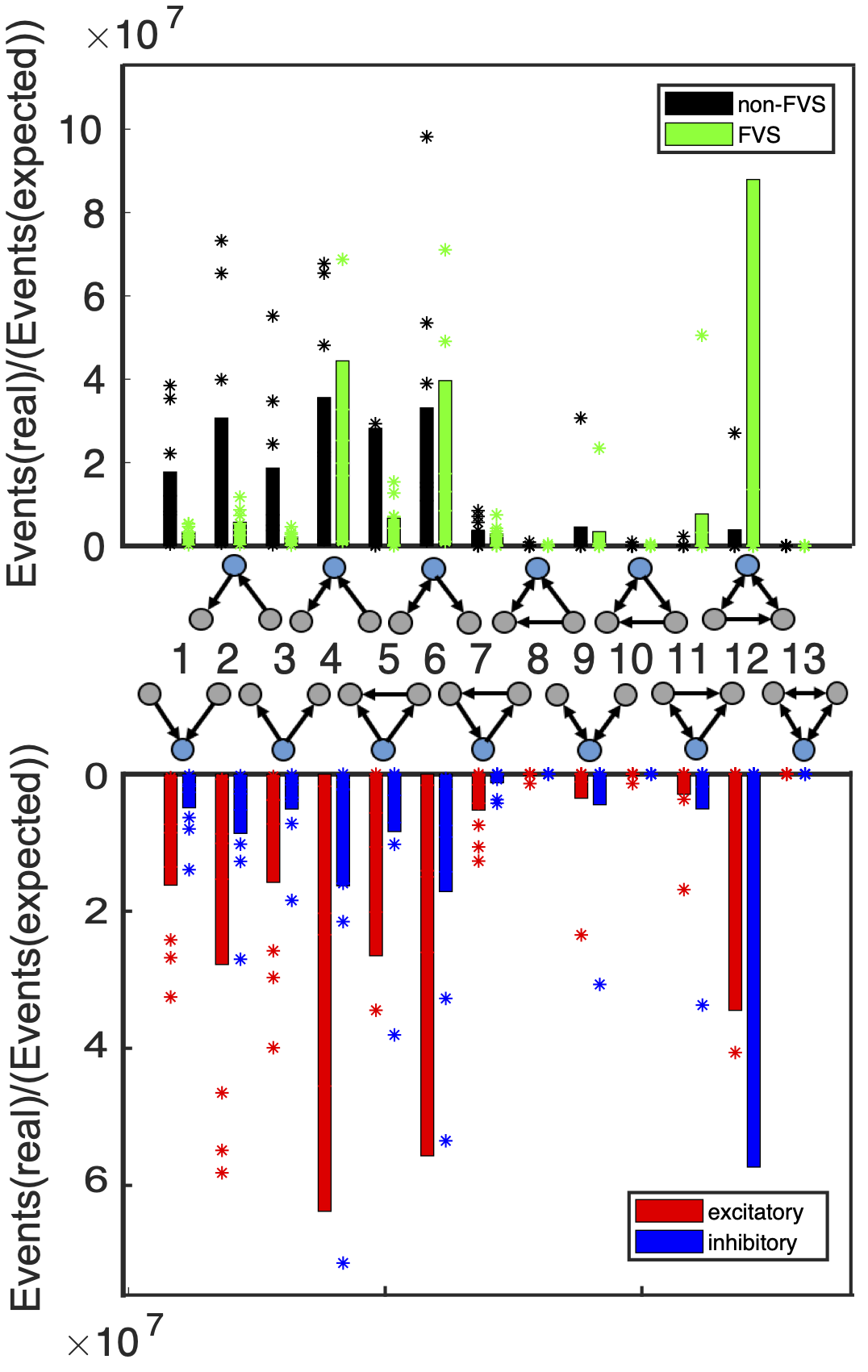

Supplement: S5 Fig — A comparison between how much more often triangle motifs could be significantly observed when comparing with expected numbers, which were calculated from the probability of non-connected or directed or bidirectional connections for pairs of nodes, for FVS of non-FVS neurons (upper panel) and excitatory and inhibitory neurons (lower panel). The connectivity density becomes higher from left to right. We could not find significant trend that FVS and inhibitory neurons have more opportunity to join in more clustered motifs (Wilcoxon signed rank test; p>0.05, FVS(motif> = 8), 9.91×10^7^±2.54×10^8^ vs. non−FVS(motif> = 8), 9.04×10^6^±1.54×10^7^; Z (7 slices)>>10), Inhibitory (motif> = 8), 4.13×10^7^±8.98×10^7^ vs. excitatory (motif> = 8), 6.69×10^6^±1.76×10^8^; Z (7 slices)>>10). The motifs were calculated using Brain Connectivity toolbox [93]. (TIFF) [file pcbi.1008846.s005.tiff]

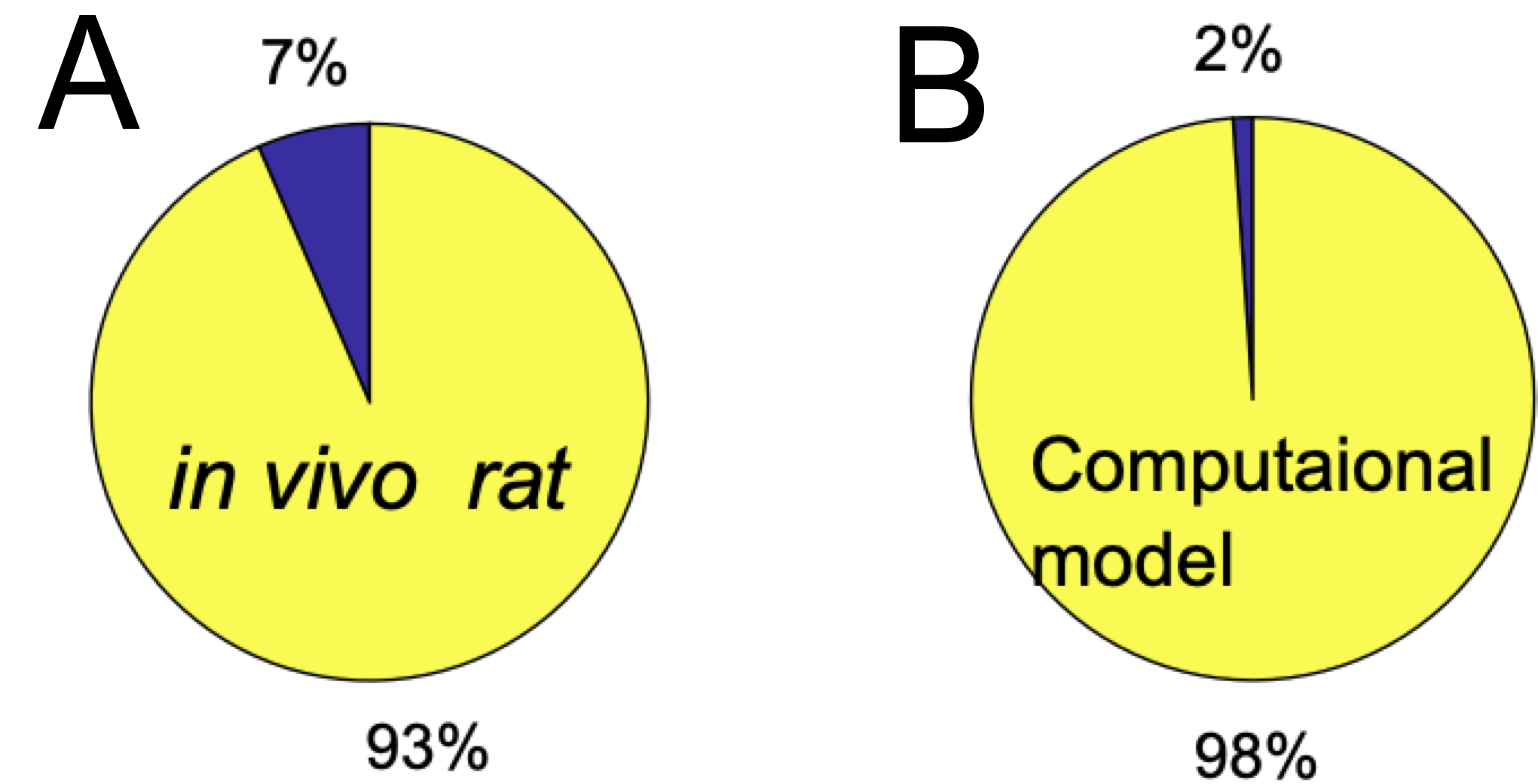

Supplement: S6 Fig — A Evaluation of inhibitory neurons in dataset recorded at rat cortex. The dataset consists of six data sets (2 probes are recorded from 3 rats). The pairs of probes are V1 and PPC, V1 and M2, M2 and PPC. We analyzed all probes independently, and compared the predicted inhibitory neurons and fast spike neurons estimated in the reference [74]. The final result is shown as median value among six data set. Refer the report in more detail about the utilized spike data. B Similar evaluation of inhibitory neurons estimated by our methods with answers in a computational model (https://github.com/Motoki878/model). Although we did not show here, prediction of excitatory neurons also showed 99% accuracy. (TIFF) [file pcbi.1008846.s006.tiff]
